# A selective low-dose cisplatin strategy enabled by melatonin and cannabinoids for targeting osteosarcoma and chondrosarcoma

**DOI:** 10.1007/s00210-026-05551-y

**Published:** 2026-06-27

**Authors:** Davut Aydın, Aylin Korkmaz Pınarbaşı, Kübra Aslan, Ali Aydın, Halit Canatan

**Affiliations:** 1 Department of Medical Biology, Institute of Health Sciences, Erciyes University, 38030 Melikgazi, Kayseri, Turkey; 2 Department of Medical Biology, Faculty of Medicine, Yozgat Bozok University, 66200 Yozgat, Turkey; 3 Department of Medical Biology, Faculty of Medicine, Erciyes University, 38030 Melikgazi, Kayseri, Turkey; 4 Genom and Stem Cell Center, Erciyes University, 38280 Talas, Kayseri, Turkey

**Keywords:** Melatonin, Cisplatin, Cannabidiol, Tetrahydrocannabinol, Osteosarcoma, Anticancer

## Abstract

Therapeutic management of osteosarcoma and chondrosarcoma is limited by cisplatin (CP)-associated systemic toxicity and acquired chemoresistance. Strategies enabling effective dose reduction while maintaining antitumor activity are urgently required. This study investigated whether melatonin (MEL) and cannabinoids (CBD, THC) enhance CP efficacy through synergistic, multi-target mechanisms in osteosarcoma (MG63, Saos2) and chondrosarcoma (SW1353) cells, with normal fibroblast (FL) and osteoblast (HC) cells as controls. Cytotoxicity and antiproliferative effects were evaluated using MTT and LDH assays. Drug interactions were quantified via combination index (CI) and dose-reduction index (DRI) analyses. Apoptotic mechanisms were assessed by Casp3, Casp8, Casp9, and Bax gene expression, DNA fragmentation, DAPI nuclear staining, and Rhodamine-123-based mitochondrial membrane potential analysis. Anti-migratory and anti-invasive effects were examined using wound-healing and invasion assays. Molecular docking was performed to predict interactions with COX-2, MMPs, ADAMTS-5, and MAPK pathway proteins. Combination regimens, particularly THC + CP + MEL, and CP + MEL, demonstrated strong synergism (CI < 1) in SW1353 and Saos2 cells, enabling up to a 2.77 - 4.38-fold reduction in CP dose. Synergistic treatments significantly upregulated apoptotic markers and induced selective DNA fragmentation, chromatin condensation, and mitochondrial depolarization in cancer cells, while normal cells showed minimal alterations. Cannabinoids markedly suppressed migration and invasion, effects further enhanced in combination treatments. Docking analyses supported high-affinity multi-target interactions consistent with the observed biological responses. Collectively, melatonin enhances cannabinoid-mediated chemosensitization to cisplatin through selective, pro-apoptotic and anti-invasive mechanisms, supporting a low-dose, multi-target combinatorial strategy for bone sarcoma therapy.

**Supplementary Information:**

The online version contains supplementary material available at https://doi.org/10.1007/s00210-026-05551-y.

## Introduction

Osteosarcoma (OS) is the most common primary malignant bone tumor in children and young adults and is characterized by highly aggressive growth, genomic instability, and a marked propensity for early pulmonary metastasis (Fathizadeh et al. 2019; Kazantseva et al. 2022). Despite multimodal treatment strategies combining neoadjuvant chemotherapy, surgical resection, and adjuvant chemotherapy, overall survival has plateaued over the past three decades. Five-year survival rates remain approximately 60–70% for patients with localized disease but drop dramatically to below 30% in metastatic or recurrent cases (Mertens and Bramwell 1994; Mirabello et al. 2009; Picci et al. 2010). This therapeutic stagnation underscores an urgent need for innovative drug development strategies targeting the molecular complexity of OS.

Cisplatin (CP) remains a cornerstone of osteosarcoma chemotherapy regimens due to its DNA crosslinking and apoptosis-inducing properties. However, its clinical utility is limited by intrinsic and acquired resistance mechanisms, including enhanced DNA repair capacity, altered apoptotic signaling, increased antioxidant defenses, and drug efflux modulation (Hattinger et al. 2021; Li et al. 2025). Furthermore, dose-dependent toxicities—particularly nephrotoxicity, ototoxicity, and neurotoxicity—restrict therapeutic escalation and compromise quality of life (Zhra et al. 2025). These limitations highlight the importance of combination strategies capable of enhancing antitumor efficacy while enabling dose reduction and toxicity mitigation.

Rational combination therapy represents a central paradigm in modern drug development, particularly for malignancies driven by heterogeneous and redundant survival pathways (Ziyadanoğulları et al. 2025). Targeting complementary mechanisms—such as oxidative stress modulation, mitochondrial dysfunction, DNA damage amplification, and apoptotic signaling—may overcome resistance and produce synergistic cytotoxic effects. In this context, bioactive phytochemicals have gained attention as adjuvant candidates due to their pleiotropic molecular actions and favorable safety profiles.

Cannabinoids, including cannabidiol (CBD) and Δ⁹-tetrahydrocannabinol (THC), exert anti-proliferative, pro-apoptotic, anti-angiogenic, and anti-migratory effects in multiple cancer models (Besser et al. 2024). Mechanistically, they modulate CB1/CB2 receptor signaling, reactive oxygen species (ROS) generation, mitochondrial membrane potential, ER stress pathways, and PI3K/Akt/mTOR signaling cascades. Emerging evidence suggests that cannabinoids can sensitize tumor cells to conventional chemotherapeutics through oxidative stress amplification and apoptosis enhancement.

Melatonin (MEL), a pleiotropic indoleamine with strong antioxidant and oncostatic properties, has also demonstrated inhibitory effects on osteosarcoma growth (Nakashima et al. 2019). MEL regulates redox homeostasis, mitochondrial function, cell cycle progression, and intrinsic apoptotic pathways, and may modulate p53 signaling and caspase activation. Importantly, melatonin exhibits context-dependent pro-oxidant activity in tumor cells, which may enhance chemotherapeutic-induced cytotoxicity while protecting normal tissues from oxidative damage.

Recent preclinical studies indicate that cannabinoids and MEL may potentiate cisplatin efficacy, reduce required CP concentrations, and attenuate chemotherapy-associated toxicity (Skórzewska and Gęca 2024; Kazantseva et al. 2021). However, the combinatorial effects of CBD, THC, and MEL together with CP in osteosarcoma models remain insufficiently characterized. The potential for multi-targeted modulation of oxidative stress, mitochondrial integrity, and apoptotic signaling in OS cells has not been systematically evaluated.

Therefore, the present study investigates the cytotoxic and combinatorial effects of CBD, THC, and MEL—administered individually and in combination with cisplatin—in osteosarcoma cell lines. By applying quantitative drug-interaction analysis and mechanistic assays, this work aims to identify synergistic interactions and provide a pharmacological rationale for developing novel multi-targeted combination strategies with improved therapeutic index in osteosarcoma.

## Material and method

### CBD and THC isolation

**Fig. 1:**
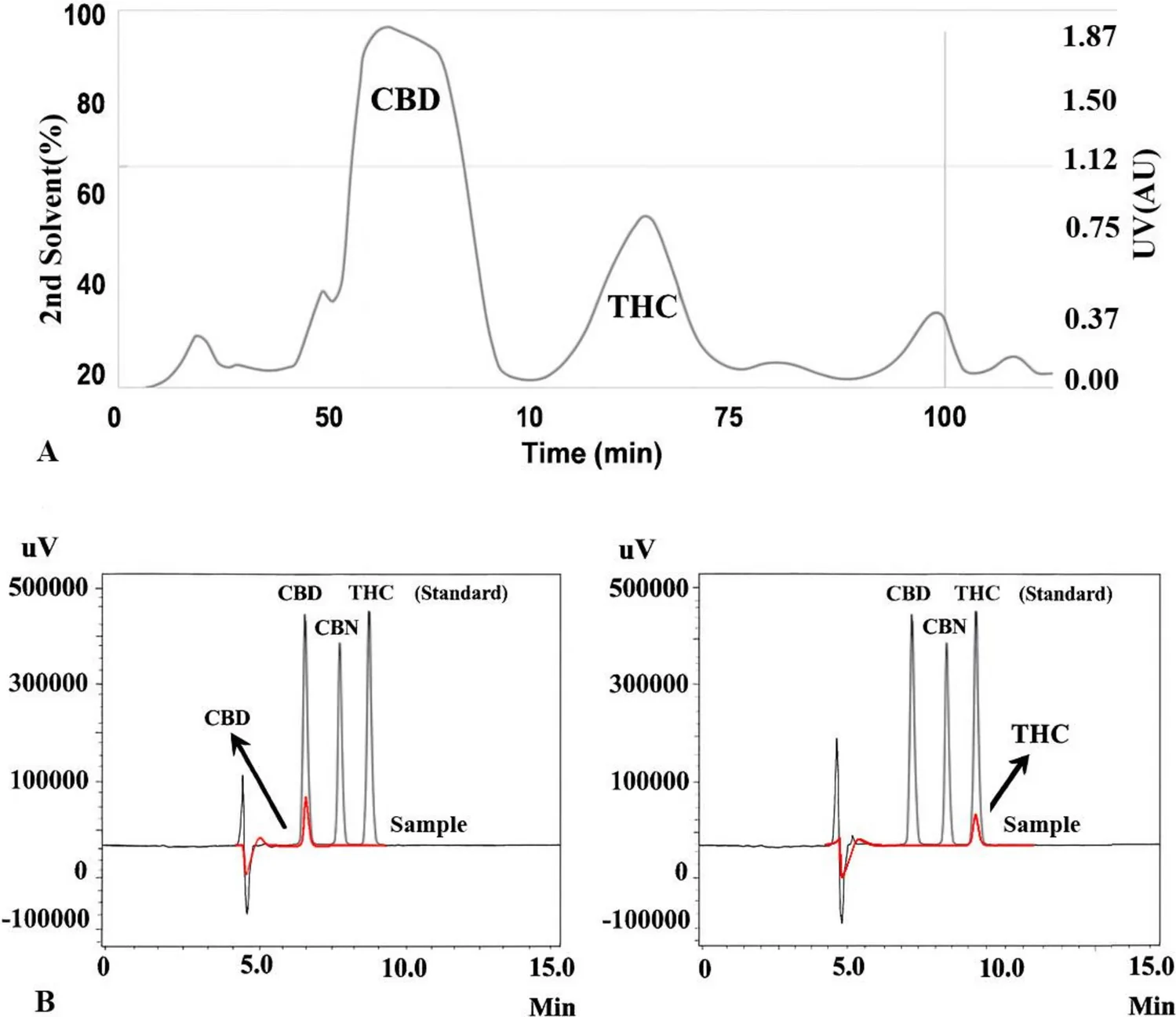
**A** Flash chromatograms of CBD and THC; 65% constant in 3 min (65% ethanol–35% water), 65–70% increase in 8 min, 70–75% increase in 3 min, 75–100% increase in 3 min, and 100% constant in 5 min. **B** HPLC chromatography of CBD, THC, and standard

Hemp flowers (Narlisaray cultivar) were obtained from the Hemp Research Institute at Yozgat Bozok University. After the hemp flowers were ground in a grinder until they became a fine powder, they were left in the oven for 1 h at 105 °C for decarboxylation. The supercritical CO_2_ extraction method (230 bar, 50 °C, and 50 kg/h cycle conditions) was employed for extraction following this process. The extract was then mixed with ethanol in a 1:1 (m/v) ratio to remove waxy structures and placed in a − 80 °C freezer for 48 h. The sample was subsequently filtered using a vacuum funnel and filter paper to separate the extracted waxes. Finally, the molecular distillation method was utilized to separate terpenes and chlorophylls. The fractions richest in CBD and THC were obtained using the molecular distillation technique at temperatures between 180 °C and 200 °C. The fractions obtained were subsequently purified with flash chromatography, C18 reverse phase cartridges (40 g, C18 column), and an ethanol–water mobile phase. In this context, the 210-nm band of the CBD and THC was determined, as shown in Fig. 1A. Purity analysis results were carried out using HPLC (2.7 µm, 4.6 × 150 mm, NexLeaf CBX, PDA detector, 210 nm, mobile phase acetonitrile:water (9:1)) as illustrated in Fig. 1B. The yield was measured as 80% and purity as 98% (Kuşçu et al. 2025).

### Detection of biological activity

#### Cancer cell lines and cell culture

Human osteosarcoma cell lines MG63 and Saos2, human chondrosarcoma cells SW1353, normal human fibroblasts (FL), and normal human osteoblasts (HC) were used in this study. All cell lines were obtained from the American Type Culture Collection (ATCC) and maintained according to ATCC recommendations. Cell line authentication was supported by the short tandem repeat (STR) profiles provided by ATCC for each cell line. Cells were cultured in Dulbecco’s Modified Eagle Medium (DMEM; Gibco, Thermo Fisher Scientific, USA) supplemented with 10% fetal bovine serum (FBS; Gibco), 100 U/mL penicillin, and 100 μg/mL streptomycin (Gibco). Cultures were maintained at 37 °C in a humidified atmosphere containing 5% CO₂. Cells were routinely passaged at 70–80% confluence using 0.25% trypsin–EDTA solution (Gibco). Experiments were performed using cells within passages p7-p10. Cell viability was assessed by the trypan blue exclusion method prior to each experiment, and only cultures exhibiting ≥ 90% viability were used. For cytotoxicity assays, cells were seeded at 7.5 × 10^3^ cells/well in 96-well plates and allowed to attach for 24 h before treatment. To ensure culture quality, all cell lines were routinely screened for mycoplasma contamination by quantitative polymerase chain reaction (qPCR) at annual intervals throughout the study period. All cultures tested negative for mycoplasma contamination before and during the experimental phase. Only authenticated, mycoplasma-free cultures were used in subsequent analyses.

### Determination of the synergy

The synergistic, additive, or antagonistic interactions among CBD, THC, MEL, and CP were evaluated using the Chou–Talalay median-effect method based on MTT assay data (Chou and Talalay 1983; Chou 2006; Mosmann 1983). Cancer cells were seeded in 96-well plates and treated for 24 h with single agents or fixed-ratio drug combinations prepared according to the ratios presented in Table 1 (Aydın et al. 2021). Each treatment was performed in triplicate and repeated in at least three independent experiments. Fraction affected (Fa) values were calculated from cell viability data and used to generate dose–response curves and median-effect plots. The median-effect parameters, including the median-effect dose (D*m*), slope (*m*), and linear correlation coefficient (*r*), were determined according to the median-effect equation. Combination Index (CI) and Dose Reduction Index (DRI) values were subsequently calculated over a range of effect levels using the Chou–Talalay equations. CI values were interpreted as synergistic (CI < 1), additive (CI = 1), or antagonistic (CI > 1) interactions according to established criteria (Chou 2006). To further visualize drug interactions, Fa–CI plots and conservative isobologram analyses were generated at ED50, ED75, and ED90 effect levels. Dose–response curves, median-effect plots, CI–Fa plots, DRI plots, and isobolograms were constructed using the calculated Fa values to provide a comprehensive assessment of combination effects. Results are presented as mean ± SD, and statistical analyses were performed as described in the Statistical Analysis section.

**Table 1:** Combination ratios used in the study

Binary	Ternary	Quaternary
CBD + MEL (16:32)	THC + CBD + MEL (12:16:32)	MEL + CBD + THC + CP (32:16:12:8)
THC + MEL (12:32)	CP + CBD + MEL (8:16:32)	
THC + CBD (12:16)	CP + THC + MEL (8:12:32)	
CP + MEL (8:32)	CP + CBD + THC (8:16:12)	
CP + CBD (8:16)		
CP + THC (8:12)		

#### Cytotoxicity test

Whether the combinations were cell cytotoxic or cytostatic was determined by the LDH method (Decker and Lohmann-Matthes 1988). Depending on the tested substances, the increase in the number of cells that died during the incubation period will cause an increase in LDH in the culture supernatant. Lactate dehydrogenase (LDH) is a stable cytoplasmic enzyme found in most cells. For this purpose, the LDH cell cytotoxicity kit was used according to the manufacturer’s procedure. Briefly, the change in the amount of formazan formed due to LDH enzyme activity was measured and evaluated according to the following formula: % Cytotoxicity = [(Substance Absorbance − Low Control/High Control − Low Control) × 100].

### Determination of the effect on the expression of apoptotic genes

To elucidate the molecular mechanism underlying the anticancer activity of the test substances, we assessed the expression profiles of five apoptosis-related genes (Caspase-3, Caspase-8, Caspase-9, Bax, and Bcl-2) in cancer cell lines treated with these substances (Pfaffl 2001; Aydın et al. 2019) (Supplemental Table 10).

#### DNA laddering test

A DNA banding test was conducted to assess the impact of compounds on DNA (Gong et al. 1994). After seeding 750,000 cells into T25 flasks and incubating for 24 h, cells were collected, washed, fixed in 70% ethanol at –20 °C, and then treated with phosphate–citrate buffer. Following centrifugation, samples were incubated sequentially with Tween-20/RNase and then proteinase K/SDS. DNA extracts and a positive control were run on a 1.5% agarose gel containing ethidium bromide, and degradation patterns were visualized (Aydın and Korkmaz, 2019).

#### DAPI-Rodamin123 staining

For DAPI-Rhodamin123 (Hirose and Komamine 1992; Emaus et al. 1986), cells were washed with DPBS and incubated with Rhodamin123 dye at 37 °C for 15 min. After this, the cells were washed with DPBS and fixed in cold methanol for 10 min. Subsequently, the cells were washed with DPBS and incubated with DAPI stain at 37 °C for 15 min. Rhodamine123 stain was visualized at 520 nm, while DAPI stain was observed at 490 nm under fluorescence microscopy (Mısır et al. 2024).

### Determination of the effect on the cell migration

A wound-healing assay was performed to assess the effects of the compounds on cell migration (Liang et al. 2007). A linear scratch was created on the cell monolayer with a p200 tip, debris was removed, and cells were washed before adding fresh medium. Test molecules were applied at their D*m* concentrations, and images of the marked scratch area were taken every 24 h until the control gap (~ 500 μm) closed (Ilhan-Ceylan et al. 2025).

### Determination of the effect on the cell invasion

Cell invasion was assessed using the CytoSelect™ 24-Well Cell Invasion Assay kit following the manufacturer’s protocol (Erkell and Schirrmacher 1988). MG63 cells (1.5 × 10^5^/chamber) were seeded in serum-free medium into ECM-coated inserts (8-µm pores), with DMEM + 10% FBS placed in the lower wells as a chemoattractant. After 6 h of incubation at 37 °C, 5% CO₂, cells that migrated to the lower membrane surface were visualized by phase-contrast microscopy and quantified from captured images.

#### Bioinformatics-supported molecular docking analyses

Designed compounds were minimized, hydrogen-completed, and optimized at the appropriate pH using Discovery Studio 2025, AutoDock4.2/Vina, and Chimera 1.19. After initial optimization in Chimera, compounds were docked against selected targets. Crystal structures retrieved from the RCSB PDB were prepared for docking, and active-site grids were generated with AutoDock4.2. Ligands were then docked into the defined pockets using Vina, and binding scores were calculated. Because cisplatin contains platinum, its geometry was optimized with Gaussian09, minimized via MolView, and converted to mol2 format with Molegro Virtual Docker. Platinum parameters (“AD4_parameters”) were updated in AutoDock4.2, torsions were set to zero, Gasteiger charges assigned, and the ligand saved in pdbqt format for docking.

## Results

### Evaluation of cell proliferation measurement

**Fig. 2:**
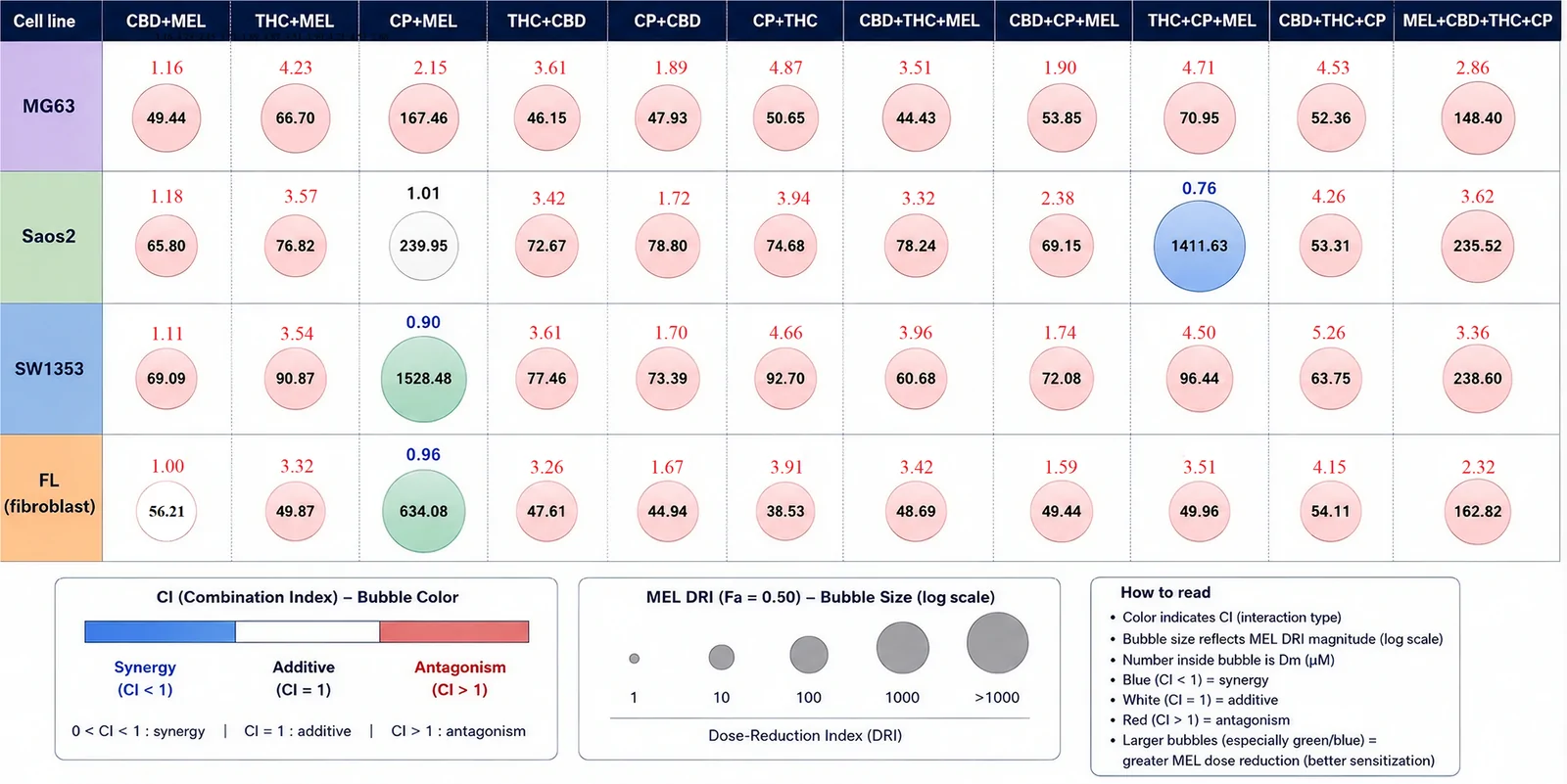
CI vs DRI integrated bubble heatmap. Bubble color: CI; bubble size DRI (Fa = 0.5); number inside bubble: D*m* (μM)

The Chou–Talalay median-effect analysis demonstrated that MEL, CBD, THC, and CP, either alone or in combination, produced distinct antiproliferative interaction profiles in MG63 and Saos2, SW1353, and FL cells. Strong linear correlations (*r* = 0.90–1.00) confirmed the suitability of the median-effect model for CI and DRI analyses (Fig. 2).

Among the single agents, CP, CBD, and THC generally showed comparable D*m*/ED50 values, whereas MEL consistently exhibited markedly weaker single-agent activity. In MG63 cells, CP displayed the lowest D*m* value (25.09 μM), followed by THC (29.91 μM) and CBD (30.66 μM), while MEL exhibited a D*m* value of 443.83 μM (Supp. Table 2) (Fig. 2). Similar patterns were observed in Saos2, SW1353, and FL cells, where MEL alone demonstrated substantially higher D*m* values than the other agents (Supp. Table 3–5). Despite its weak monotherapy activity, MEL markedly enhanced the efficacy of several combinations. In MG63 cells, CBD + MEL reduced the D*m* value to 49.44 μM, while CBD + THC + MEL showed one of the strongest cooperative effects with a D*m* value of 44.43 μM (Supp. Table 2) (Fig. 2). Similar improvements were observed in Saos2 and SW1353 cells, where MEL-containing combinations substantially decreased effective dose requirements (Supp. Table 3 and 4). However, the THC + CP + MEL combination in Saos2 cells displayed an unusually low D*m* value (13.34 μM), suggesting a potentially synergistic interaction under the tested conditions (Supp. Table 3) (Fig. 2).

DRI analyses further confirmed the therapeutic advantage of MEL-containing combinations. In MG63 cells, CBD + MEL and CBD + THC + MEL yielded MEL DRI values of 80.92 and 117.69, respectively (Supp. Table 6). In Saos2 cells, MEL-associated DRI values were lower but remained favorable, particularly in CBD + MEL (6.33) and CBD + THC + MEL (6.44) (Supp. Table 7) (Fig. 2). SW1353 data also demonstrated considerable MEL dose-reduction effects, although less pronounced than previously observed, with MEL DRI values reaching 30.22 for CBD + MEL, 32.80 for CBD + THC + MEL, and 32.21 for CBD + CP + MEL (Supp. Table 8). Similarly, high MEL DRI values were also detected in FL fibroblasts, especially in CBD + THC + MEL (139.20) and CBD + CP + MEL (139.45) (Supp. Table 9) (Fig. 2).

Conservative isobologram analyses supported these findings, showing that several binary combinations localized below the theoretical additive lines at ED50, ED75, and ED90 levels, indicating synergistic interactions between MEL-, cannabinoid-, and CP-based regimens (Supp. Figure 1–4). However, some combinations approached or crossed the additive boundaries, suggesting that the magnitude of synergism depended on both cellular background and combination composition. According to the classical Chou–Talalay CI classification system, most combinations demonstrated additive to mildly antagonistic interactions rather than strong synergism (Supp. Table 1) (Fig. 2). In MG63 cells, no pronounced synergistic interaction was observed, although several regimens approached the light or moderate antagonistic threshold. In Saos2 cells, the THC + CP + MEL combination exhibited the most notable cooperative interaction (CI = 0.76), corresponding to moderate synergy; however, its simultaneously obtained D*m* value suggested limited biological potency despite the mathematical indication of synergy. In SW1353 cells, the CP + MEL combination remained near the boundary between light synergy and additivity (CI = 0.90), whereas the remaining combinations were predominantly additive or mildly antagonistic. No clearly synergistic CI profile was detected in FL normal fibroblasts (Fig. 2).

Collectively, these findings indicate that biologically meaningful therapeutic cooperation may occur even when CI values remain within the additive range. Therefore, the therapeutic benefit of these combinations appears to arise predominantly from multi-targeted biological cooperation and substantial dose-reduction capacity rather than from strict mathematical synergism alone.

### Evaluation of cytotoxic activity of test molecules

**Fig. 3:**
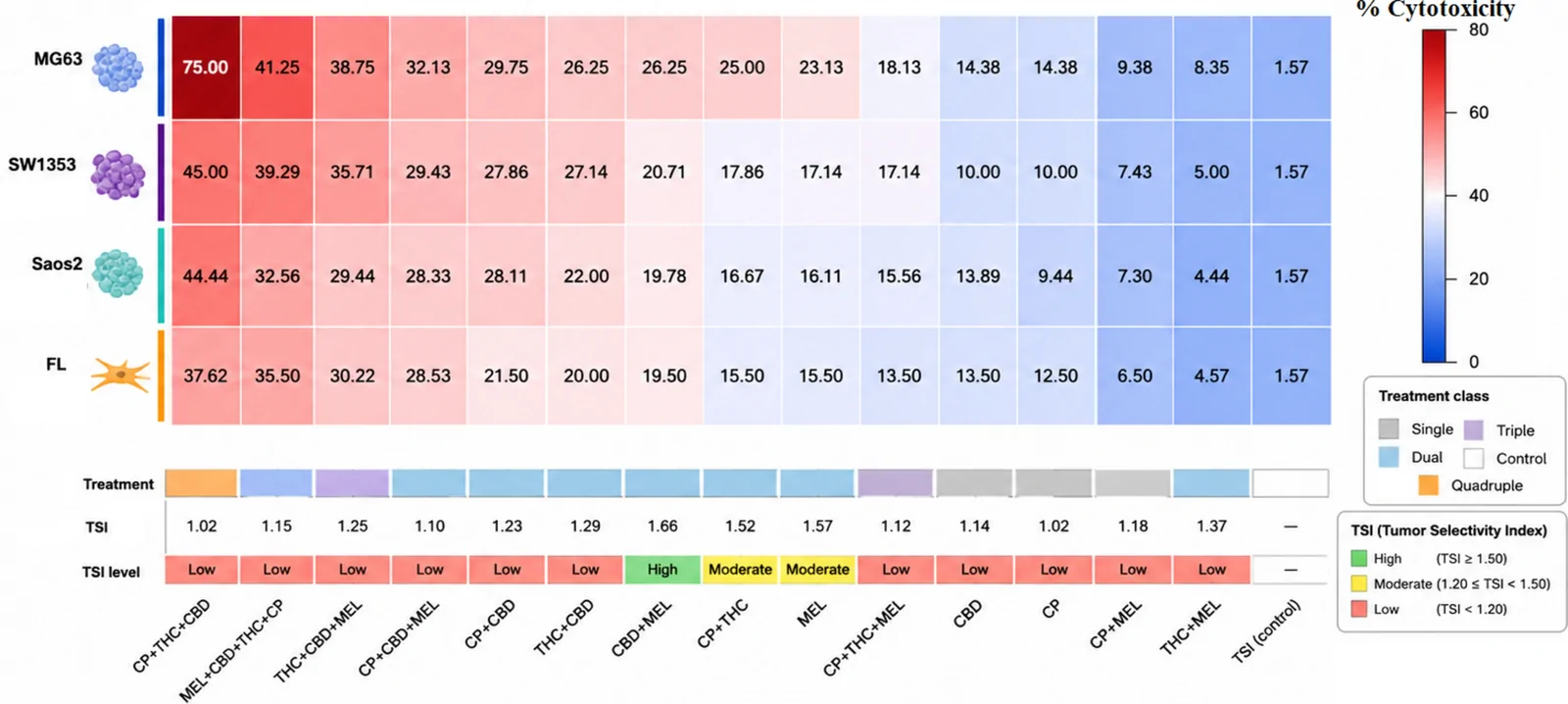
% Cytotoxicity values of test substances at D*m* concentrations

One objective of this study was to evaluate the effects of test molecules and combinations on membrane integrity and to determine their cytotoxic activities. The release of cytoplasmic LDH from damaged plasma membranes can be considered a marker for cell cytotoxicity. Thus, cell cytotoxicity can be indirectly estimated by measuring the leaked LDH released into the medium using a cytotoxicity kit. Based on the absorbance results, the individual test molecules induced cytotoxicity ranging from 9.45 to 20.0% in the FL normal cell line, while they induced cytotoxicity ranging from 9.44 to 29.44% in bone cancer cell lines (MG63, Saos2, and SW1353) (Supp. Table 11) (Fig. 3).

When the binary combinations were examined, the cytotoxicity rates of THC + MEL and CP + MEL combinations in bone cancer cell lines ranged from approximately 4.44–9.38% and in FL normal cells ranged from 4.57 to 6.50%, which are within ideal limits (Supp. Table 11) (Fig. 3). The binary combinations of CBD + MEL and CP + THC exhibited a cytotoxic effect similar to the control (FL cell line, 13.50%) in Saos2 (14.44%) and SW1353 (17.14%) cells, respectively. Of the triple combinations, the cytotoxicity rates of CP + THC + MEL combinations in bone cancer cell lines ranged from approximately 16.11–18.13% and in FL normal cells ranged from 15.50%, which are within ideal limits (Supp. Table 11) (Fig. 3).

When the cytotoxicity results of the double and triple combinations were examined, the findings indicated that the combination products reduced the cytotoxic effect on normal FL cells compared to cancer cells. When we evaluated the MTT method, which demonstrates antiproliferative activity, and the LDH method, which reveals cytotoxic properties, together, we can say that the new combinations exhibited the most optimal antiproliferative and cytotoxic effects against normal cells (Supp. Tables 2–5) (Fig. 3). This means that the new combinations at the D*m* concentration were sufficiently toxic against bone cancer cell lines but safe against FL cells, providing an opportunity for further preclinical studies. Furthermore, when examining the Tumor Specificity Index (TSI), the double combinations demonstrated greater selectivity against tumor cells (Fig. 3).

### Gene expression analysis results of test items

**Fig. 4:**
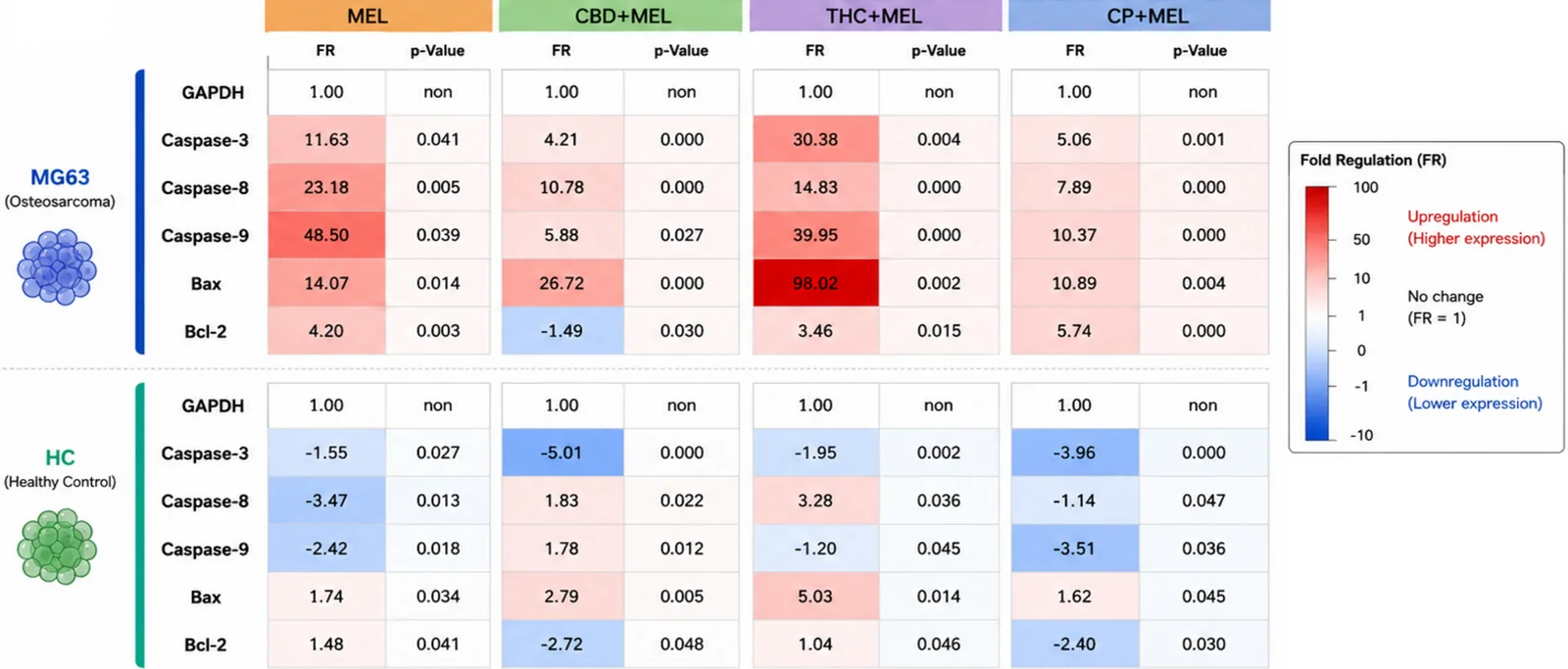
Expression levels of 5 genes in cells treated with MEL, CBD + MEL, THC + MEL, and CP + MEL

To understand the anticancer mechanism of the MEL, CBD + MEL, THC + MEL, and CP + MEL combination, we determined the expression profile of a total of 5 genes related to apoptosis in MG63 cancer cell lines, as well as HC normal cell lines (Supp. Table 12) (Fig. 4). According to gene expression analyses, MEL and THC + MEL combination caused highly upregulation of Casp3, Casp8, Casp9, and Bax genes in the MG63 cancer cell line (Supp. Table 12) (Fig. 4).

CBD + MEL combination caused highly upregulation of Casp8 and Bax genes in the MG63 cancer cell line (Supp. Table 12) (Fig. 4). CP + MEL combination caused middle upregulation Casp9 and Bax in the MG63 (Supp. Table 12) (Fig. 4). In normal HC cells, MEL and combinations caused only a little downregulation of these gene (Supp. Table 12) (Fig. 4). This situation explains that the combinations show its effect by triggering vigorously apoptotic gene upregulation. In normal cells, the combinations show opposite effect, although more limited. This situation is consistent with the MTT and LDH tests of combinations and is due to its low toxic effect. Gene expression analysis results showed that the combinations have a greater apoptotic effect, which is more pronounced and selective in its action on cancer cells.

### Determination of DNA banding potential of test substances

**Fig. 5:**
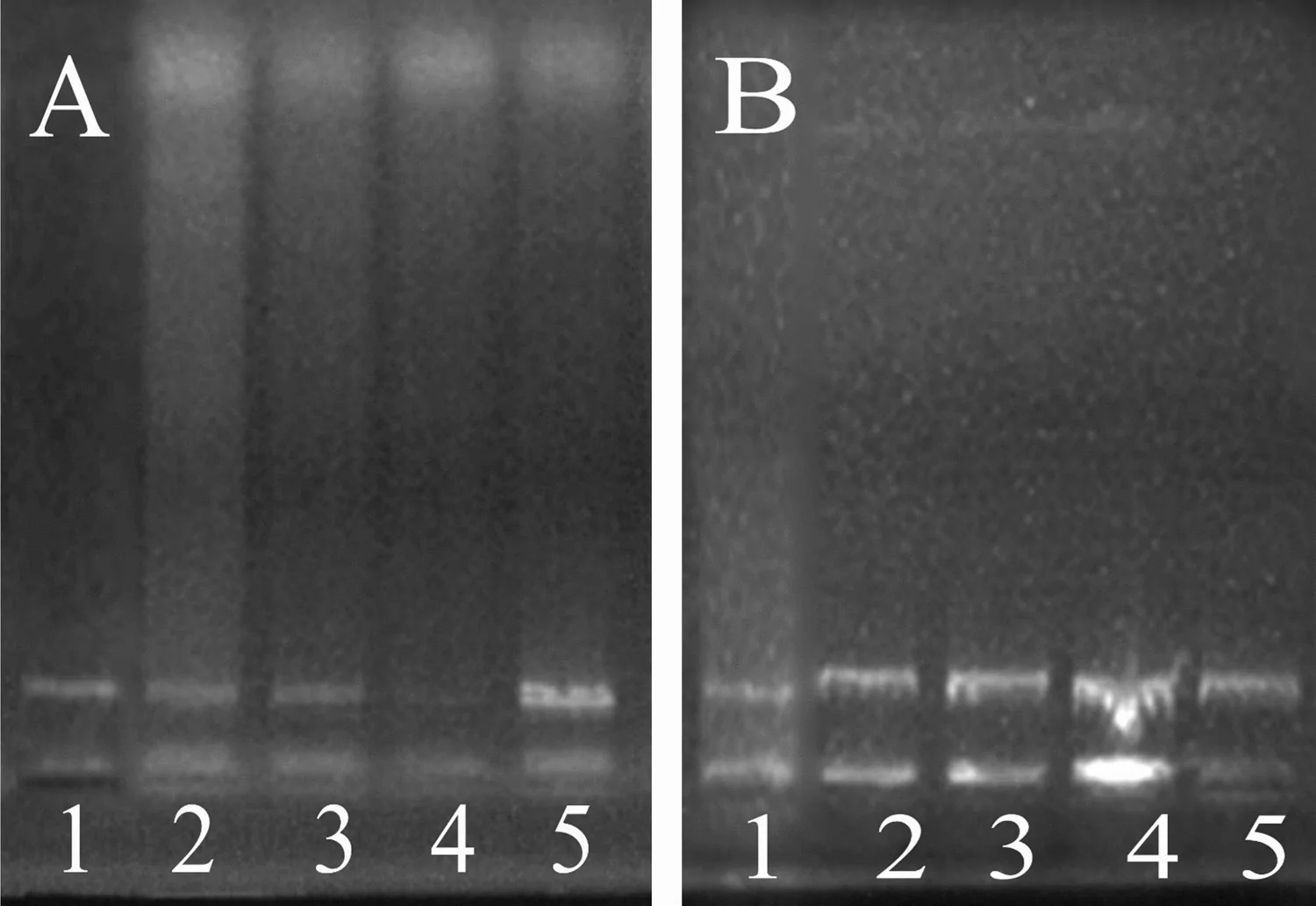
Effects of tested combinations on DNA fragmentation in MG63 **A** bone cancer cells and HC **B** bone normal cells (1. Control, 2. CBD + MEL, 3. THC + MEL, 4. CP + MEL, 5. MEL)

DNA banding assays were performed using MG63 and HC cells to determine the DNA degradation activity of the tested combinations, an indicator of apoptosis, at D*m* (IC50) concentrations. According to the DNA banding assay, no DNA degradation was observed in the controls (well 1), while the combination resulted in highly visible DNA banding in the wells (wells 2–5) (Fig. 5).

Furthermore, when comparing the cancer cell (A) to the control cell (B), less DNA degradation was observed in the control cell. Therefore, it can be strongly suggested that one of the mechanisms of action of the CBD + MEL, THC + MEL, and CP + MEL combinations that caused DNA banding in MG63 cancer cells may be related to the apoptosis death pathway and that they act in a cancer-specific manner.

### Evaluation of fluorescent staining results of test items

**Fig. 6:**
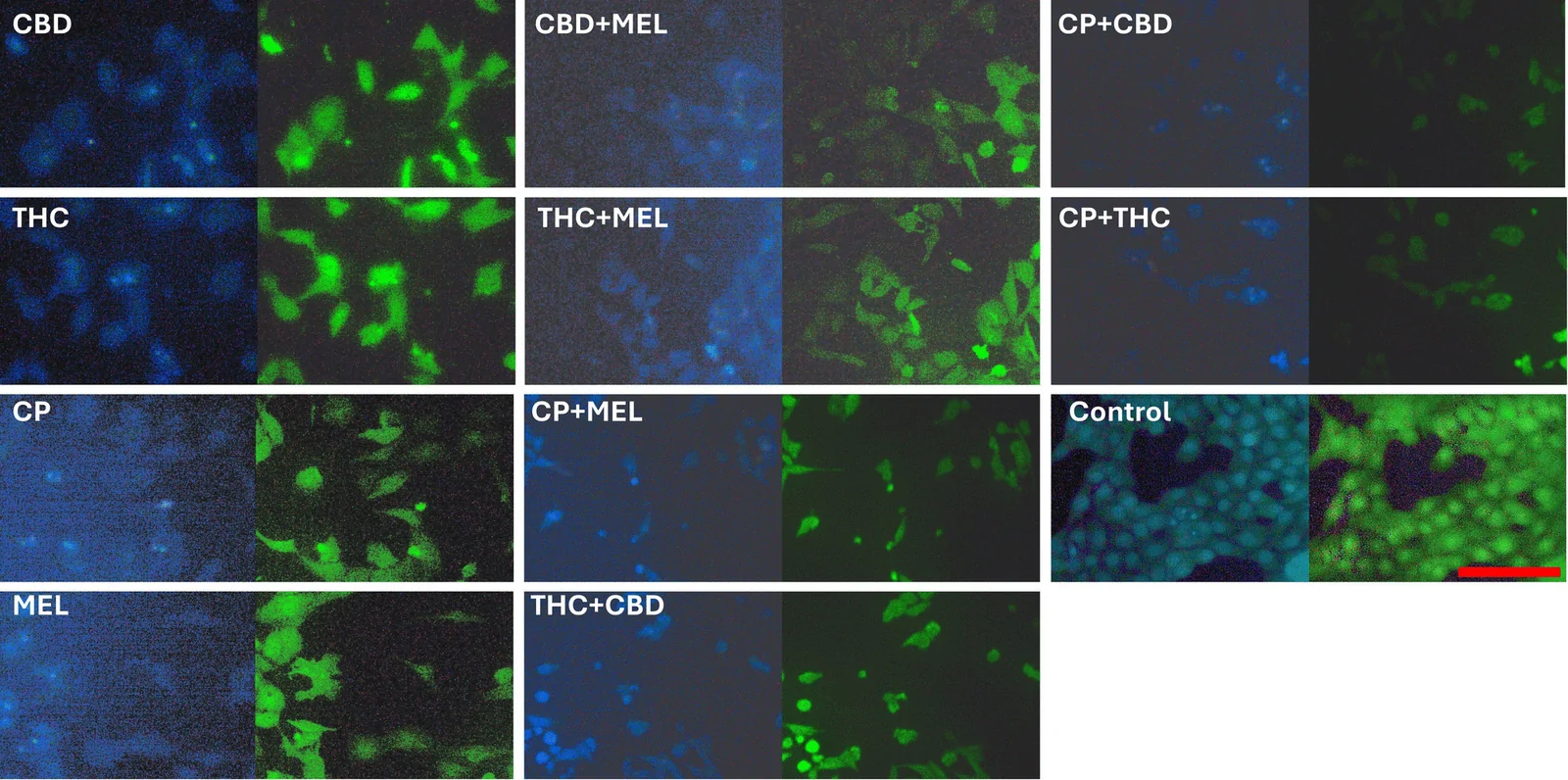
DAPI/Rhodamine 123 staining in MG63 cancer cell lines. All scales are 100 µm

To determine the effects of the single and combinations molecules on mitochondrial membrane potential and cell death, MG63, Saos2, and SW1353 cancer cell lines, along with FL normal cell lines, were stained with DAPI (blue) and Rhodamine123 (green) fluorescent dyes and examined under a fluorescence microscope. DAPI staining indicated that the single and combinations molecules increased cell death more than the single and combinations molecules (the nucleus was intensely stained blue in the treated cells) (Fig. 6) (Supp. Figure 5–7). Rhodamine123 staining also demonstrated that the single and combinations molecules caused a more significant decrease in mitochondrial membrane potential than the single and combinations molecules (intensity decreased in treated cells, while it remained intensely green in the control) (Fig. 6) (Supp. Figure 5–7). In summary, an increase in cell death and a decrease in mitochondrial membrane potential were observed after applying the single and combinations molecules to cancer cells.

### Effect of test substances on cell migration

**Fig. 7:**
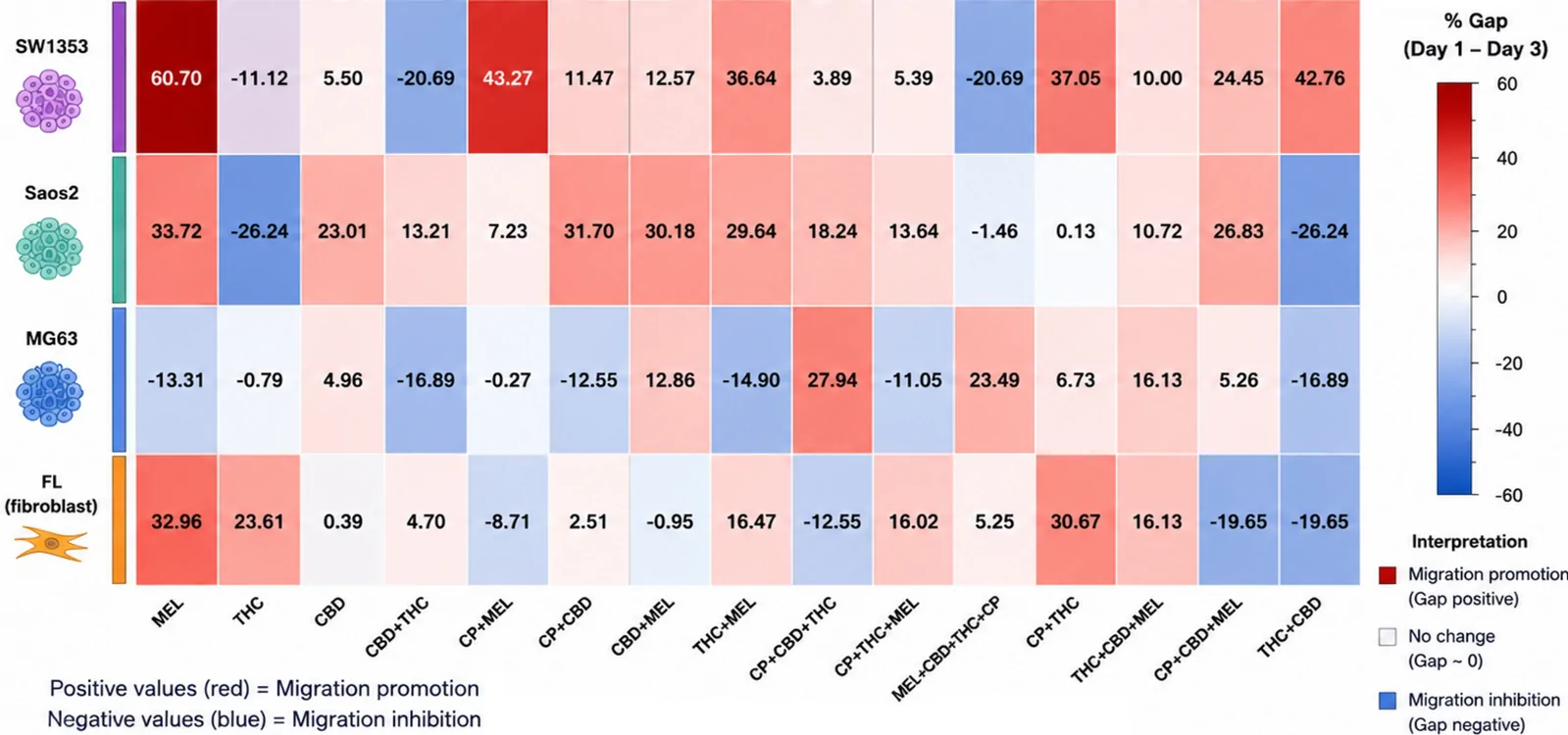
Results of migration % area analysis of the cells using ImageR software

Migratory capacity is a key characteristic of cancer cells and is a target for new anticancer agents. Cancer cells possessing migratory capacity can evade apoptosis. Therefore, one goal of newly developed anticancer drugs is to significantly reduce this migratory capacity of cancer cells. According to a time-dependent migration test, the tested combinations significantly suppressed the migratory capacity of MG63, Saos2, and SW1353 cancer cells compared to control cells (Supp. Figures 8–13) (Fig. 7). Compared to the control, these substances used at D*m* concentrations significantly reduced the migration rate in the cell lines, as seen in Supp. Figures 8–13. When we increased the incubation time during this test, it was observed that the control group continued to grow in clumps, typical of cancer cells, while in the test compound-treated groups, the space within the insert was not filled and the cells shrank. Migration, along with angiogenesis and invasion, is a crucial step in metastasis. Therefore, the migration inhibition capacity of the test substance combinations used directly reflects their metastasis inhibition capacity. Based on these test results, the antimetastatic effect is thought to be responsible for some of the anticancer activity of the test substances because these combinations inhibit cell migration (Fig. 7). When area analysis was performed with ImageR, it was seen that the combinations of CP + MEL, CP + CBD + MEL, and CP + CBD + THC in FL cells between the 1 st and 3rd days of migration inhibited cell migration by approximately 10% (Supp. Table 13) (Fig. 7). In the MG63 cell line, the combinations of MEL, THC + MEL, THC + CBD, CP + CBD, THC + CBD + MEL, and CP + THC + MEL inhibited cell migration by 10% (Supp. Table 13). In the Saos2 cell line, only THC inhibited cell migration by 10% (Supp. Table 13). In the SW1353 cell line, the combinations of CBD and MEL + CBD + THC + CP inhibited cell migration by 10% (Supp. Table 13) (Fig. 7). Compared to the control, it was found that the other combinations slowed cell migration although they did not stop it.

### Effect of test substances on cell invasion

**Fig. 8:**
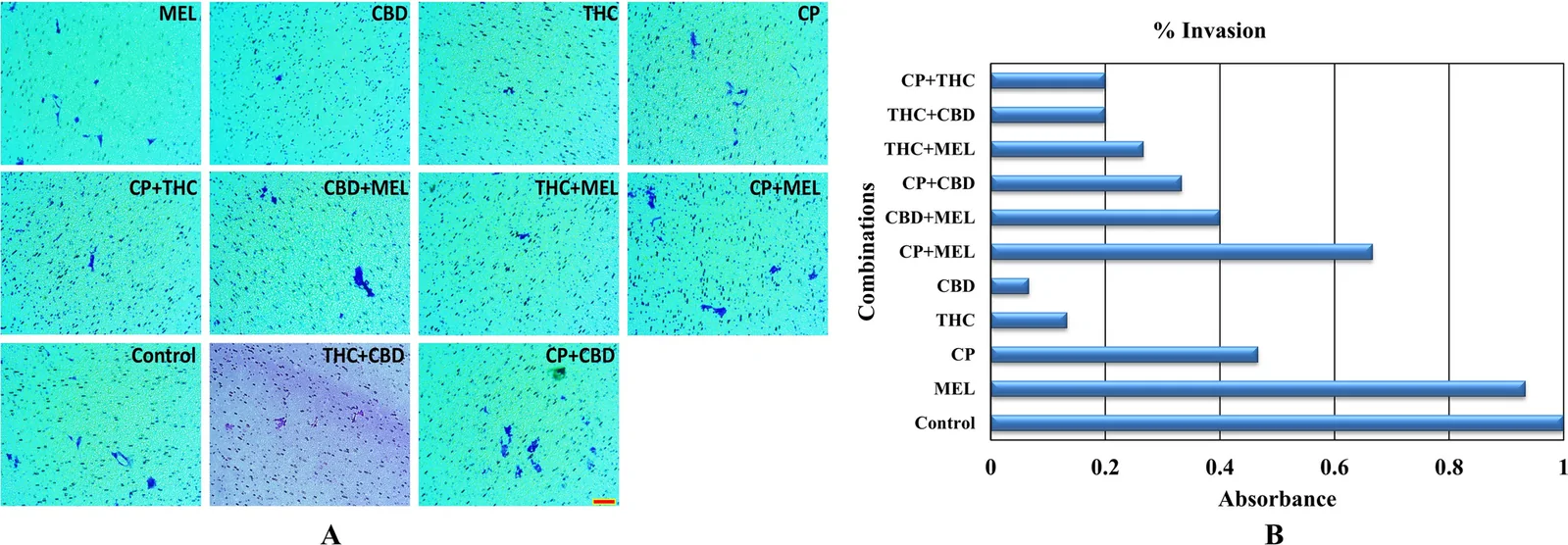
Demonstration of the effects of the tested combinations on the invasion of the MG63 cancer cell line using cell invasion measurement (**A**). Absorbance values of stained invasive cells measured at 560 nm (**B**). Bar 50 µm

When the test molecules were examined for their ability to inhibit invasiveness in the MG63 cell line, CBD and THC cannabinoids were the most effective inhibitors, with 93% and 86% inhibition (Fig. 8). When the effects of CP and MEL molecules on invasiveness were examined, inhibition values ​were 53% and 6%, respectively. When combinations were examined, CP + MEL caused 33% inhibition, CBD + MEL 60%, CP + CBD 66%, THC + MEL 73%, THC + CBD 80%, and CP + THC 80%. Consistent with the migration data, MEL and THC administration also reduced the degree of inhibition in the invasion data.

### Effect of test substances on the morphology of cells

Some morphological changes were observed in MG63, Saos2, and SW1353 cells after 24-h incubation with the tested combinations at their D*m* concentrations. These combinations induced a transition from asteroid to globular shape, reduced substrate adhesion, bubble/spike/bleb formation, cell or cytoplasmic shrinkage, apoptotic pockets, and in some cases complete disintegration (Supp. Figures 14–18). These degenerative changes and the reduction in adherent cell numbers increased proportionally with concentration. While globular structures in control cells reflected normal division, those in treated cells indicated apoptosis. Low-dose morphological alterations were consistent with the antiproliferative activity of the compounds. Individually, CBD and CP caused marked morphological disruption (Supp. Figures 14–18). In combination, however, THC and MEL appeared to moderate the destructive effects of CBD and CP (Supp. Figures 14–18), aligning with earlier findings: THC reduces CBD-induced cytotoxicity, and MEL mitigates CP toxicity, thereby broadening the effective dose range.

### Molecular docking results

Computer-aided drug design approaches are widely used to facilitate the identification and prioritization of potential ligand–target interactions during early-stage drug discovery. In the present study, molecular docking was employed as an exploratory in silico tool to investigate the potential binding of THC, CBD, melatonin, and cisplatin to selected proteins associated with cancer-related signaling pathways. The calculated docking scores and interaction parameters are summarized in Table 2.

**Table 2:** Docking scores of THC, CBD, melatonin, and cisplatin

Target	PDB ID	Compounds	Docking score	Target	PDB ID	Compounds	Docking score
Erk2	4G6N	THC	− 3.776	MMP-9	6ESM	THC	− 4.576
		CBD	− 3.573			CBD	− 6.924
		Melatonin	− **7.429**			Melatonin	− 6.857
		Cisplatin	− 4.390			Cisplatin	− 2.180
Actin	3HBT	THC	− 2.076	ADAMTS-5	6YJM	THC	− 6.175
		CBD	− 4.499			CBD	− **7.006**
		Melatonin	− 3.853			Melatonin	− 6.526
		Cisplatin	− 4.590			Cisplatin	− **7.010**
IkB	4KIK	THC	− 4.807	GAPDH	6IQ6	THC	− 2.924
		CBD	− 5.917			CBD	− 3.051
		Melatonin	− 6.694			Melatonin	− 3.062
		Cisplatin	− 3.540			Cisplatin	− 4.670
COX-2	5IKV	THC	− **8.878**	JNK2	3E7O	THC	− 6.564
		CBD	− 5.892			CBD	− 6.574
		Melatonin	− 6.874			Melatonin	− **7.967**
		Cisplatin	− 4.750			Cisplatin	− 3.340
iNOS	4NOS	THC	0.351	PGE2	3WFH	THC	− 3.750
		CBD	− 3.008			CBD	− 3.872
		Melatonin	− 3.817			Melatonin	− 3.569
		Cisplatin	− 3.590			Cisplatin	− 4.320
MMP-3	4G9L	THC	− 6.937	β-catenin	3SL9	THC	− 2.114
		CBD	− **7.379**			CBD	− 2.185
		Melatonin	− 6.543			Melatonin	− 3.439
		Cisplatin	− 5.710			Cisplatin	− 3.590

Among the evaluated interactions, THC exhibited the most favorable docking score with COX-2 (− 8.878), while CBD showed relatively strong predicted binding affinities toward MMP-3, MMP-9, and ADAMTS-5 (docking scores ≤ − 7). Melatonin demonstrated favorable docking scores with JNK2 (− 7.967) and ERK2 (− 7.429), whereas cisplatin showed a comparatively strong predicted interaction with ADAMTS-5 (− 7.010). These findings suggest that the investigated compounds may possess the capacity to interact with multiple cancer-associated proteins at the molecular level.

However, it should be emphasized that molecular docking provides only computational predictions of potential binding poses and relative binding affinities. Therefore, these results should be considered hypothesis-generating observations rather than evidence of target engagement, pathway modulation, or mechanism of action. Experimental studies are required to validate whether the predicted interactions occur under biological conditions and contribute to the pharmacological effects observed in the present study.

## Discussion

The present study demonstrates that MEL, CBD, THC, and CP exert significantly enhanced anticancer activity when administered in rational combinations against osteosarcoma and chondrosarcoma models. From a drug development standpoint, the most notable finding is the robust pharmacodynamic synergy observed in both binary and ternary regimens, particularly THC + CP + MEL, and CP + MEL combinations. Although MEL required relatively higher concentrations to induce cytotoxicity as a single agent, its incorporation into combinatorial regimens markedly amplified overall efficacy, confirming its function as a biological response modifier rather than a primary cytotoxic compound. This is consistent with prior evidence indicating that MEL enhances chemo- and radiosensitivity through modulation of redox signaling, mitochondrial integrity, and apoptosis-related pathways (Talib et al. 2021). According to the Chou–Talalay CI classification, most combinations exhibited additive rather than strong synergistic interactions. In MG63 cells, several regimens approached additive, although no pronounced synergistic profile was observed. In Saos2 cells, the THC + CP + MEL combination showed the most notable cooperative interaction (CI = 0.76), consistent with moderate synergy; however, its simultaneously obtained D*m* value (13.34 μM) suggested limited biological potency despite the mathematical indication of synergy. In SW1353 cells, CP + MEL remained near the light synergy/additive boundary (CI = 0.90), while the remaining combinations were predominantly additive or mildly antagonistic. No clearly synergistic CI profile was detected in FL normal fibroblasts.

Despite these predominantly additive CI profiles, MEL-containing combinations produced notable DRI advantages, particularly in MG63, Saos2, and SW1353 cells. CBD + MEL and CBD + THC + MEL markedly reduced the effective MEL dose, while SW1353 data still demonstrated considerable MEL dose-reduction effects, indicating biologically meaningful sensitization despite the absence of strong CI-defined synergism. Importantly, significant DRI responses were also observed in FL cells, suggesting that these combinations remain biologically active in non-cancerous cells. Collectively, these findings suggest that the therapeutic potential of the tested regimens may derive primarily from enhanced dose-reduction capacity and multi-targeted biological cooperation rather than from classical strong synergism alone, highlighting the need for further studies on selectivity, mechanistic pathways, and in vivo safety.

Apoptosis emerged as the central mechanistic axis underlying the observed synergy. The coordinated upregulation of Casp3, Casp8, Casp9, and Bax, combined with DNA fragmentation, chromatin condensation (DAPI staining), and Rhodamine-123-detected mitochondrial depolarization, indicates activation of both intrinsic and extrinsic apoptotic cascades. Importantly, these effects were pronounced in malignant cells but minimal in normal counterparts, reinforcing the concept of cancer-selective vulnerability. Similar cannabinoid-mediated apoptotic responses have been documented in breast, prostate, and glioblastoma models, where mitochondrial dysfunction and caspase activation serve as primary execution pathways (Chakravarti et al. 2014). In the context of osteosarcoma—characterized by genomic instability and altered apoptotic thresholds—multi-pathway activation may overcome resistance mechanisms linked to p53 dysfunction and enhanced DNA repair capacity.

Beyond cytotoxicity, the pronounced suppression of migration and invasion further strengthens the translational relevance of these combinations. CBD alone reduced invasion by more than 90% in MG63 cells, and combinatorial regimens potentiated this anti-metastatic effect. Given that pulmonary metastasis remains the primary cause of mortality in osteosarcoma, targeting metastatic competence is as critical as inducing tumor cell death. Cannabinoids have been reported to downregulate MMP-9 expression, inhibit epithelial–mesenchymal transition (EMT), and suppress cytoskeletal remodeling in breast and lung cancer models (Ramer et al. 2012). The strong anti-invasive phenotype observed here suggests that MEL–cannabinoid–CP combinations may impair both tumor survival and metastatic dissemination.

In silico docking analyses provide further mechanistic coherence to these biological observations. THC demonstrated high binding affinity toward COX-2, a pro-inflammatory enzyme frequently overexpressed in osteosarcoma and associated with aggressive behavior. CBD showed strong predicted interactions with MMP-3, MMP-9, and ADAMTS-5—key mediators of extracellular matrix degradation—while MEL exhibited affinity for MAPK pathway proteins (ERK2, JNK2), central regulators of stress and apoptotic signaling. CP interaction with ADAMTS-5 further suggests potential cooperative modulation of extracellular matrix remodeling (Mihanfar et al. 2022; Yasukawa et al. 2016). Together, these target profiles support a multi-targeted pharmacological framework in which DNA damage induction (CP), redox modulation (MEL), mitochondrial destabilization (CBD/THC), and invasion suppression converge to produce synergistic antitumor activity.

From a drug development perspective, the integration of phytocannabinoids and melatonin into cisplatin-based regimens represents a rational repurposing strategy aimed at increasing therapeutic index rather than merely cytotoxic intensity. The observed synergy, dose reduction potential, cancer selectivity, and anti-metastatic activity collectively justify further investigation in in vivo osteosarcoma models, including pharmacokinetic interaction studies and toxicity profiling. In particular, evaluating whether MEL mitigates cisplatin-induced nephrotoxicity while preserving antitumor synergy would be of high translational value.

### Mechanistic model

**Fig. 9:**
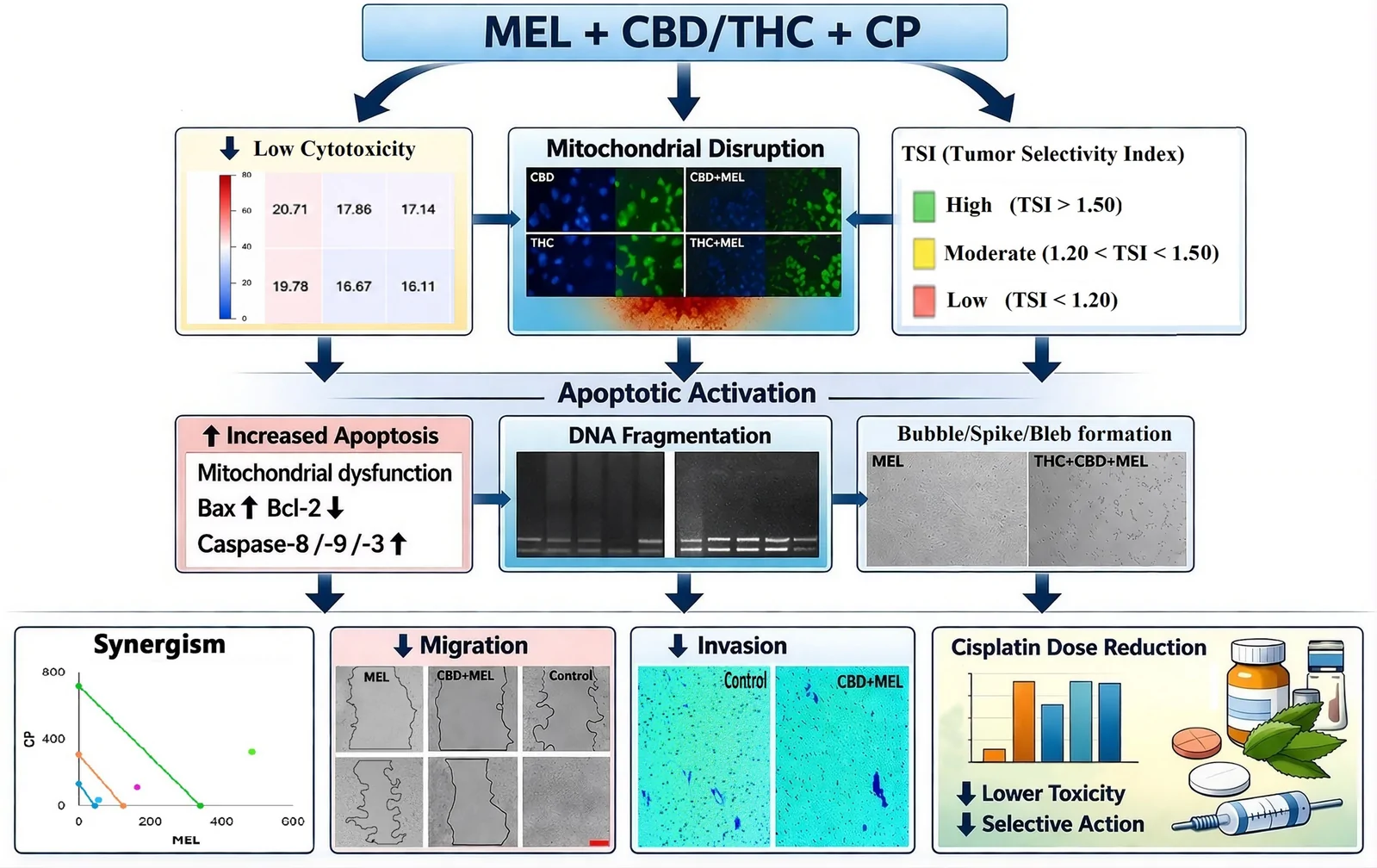
Mechanistic model illustrating the synergistic anticancer effects of MEL, CBD/THC, and CP in osteosarcoma and chondrosarcoma cells

Based exclusively on the experimental findings obtained in the present study, MEL-containing cannabinoid and cisplatin combinations appear to reduce cancer cell viability, induce mitochondrial dysfunction, activate apoptotic pathways, promote DNA fragmentation and apoptotic morphology, and suppress migration and invasion (Fig. 9). Importantly, these effects were accompanied by substantial dose-reduction advantages and favorable tumor selectivity.

## Conclusion

In conclusion, this study demonstrates that melatonin–cannabinoid–cisplatin combinations offer a promising therapeutic approach for osteosarcoma and chondrosarcoma. These combinations enhanced apoptosis, inhibited migration and invasion, reduced cisplatin requirements, and spared normal cells. Their synergistic, multi-targeted effects help overcome key limitations of standard chemotherapy. Further in vivo and clinical studies are needed to confirm their potential for future bone cancer treatments.

## Supplementary Information

Below is the link to the electronic supplementary material.

Supplementary file1 (DOCX 6.44 MB)

Supplementary file2 (PDF 123 kb)

Supplementary file3 (PDF 122 kb)

Supplementary file4 (PDF 123 kb)

Supplementary file5 (PDF 123 kb)

## Data Availability

All source data for this work (or generated in this study) are available upon reasonable request.
