# Supplementary material for: A selective low-dose cisplatin strategy enabled by melatonin and cannabinoids for targeting osteosarcoma and chondrosarcoma
Source: Naunyn Schmiedebergs Arch Pharmacol. 2026 Jun 27;399(12):19709–22. doi: 10.1007/s00210-026-05551-y (PMC13593660; doi:10.1007/s00210-026-05551-y)
Supplement: Supplementary file 4 [file 210_2026_5551_MOESM5_ESM.pdf]

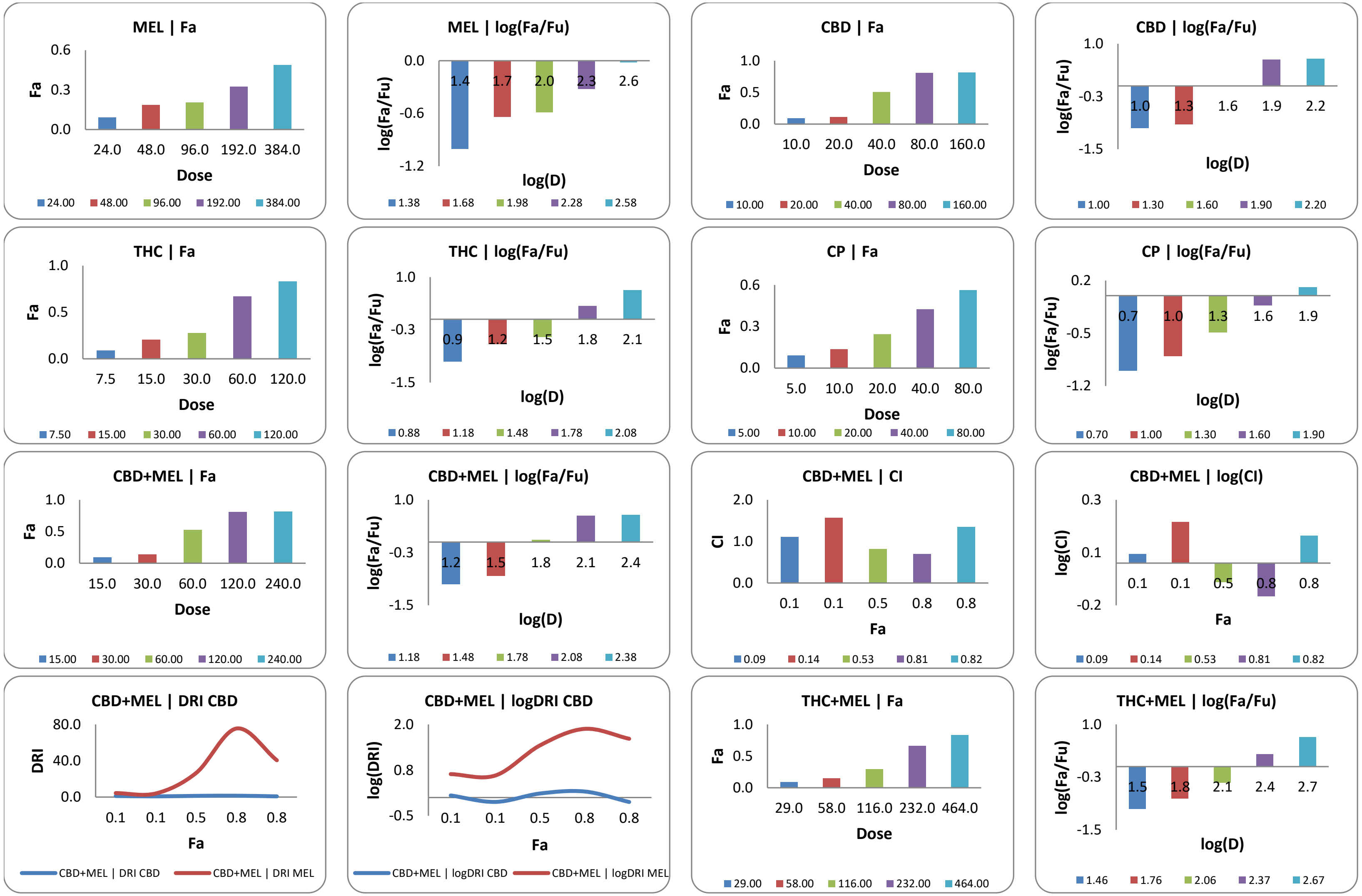
MEL | Fa
Fa
0.6
0.3
0.0
24.0 48.0 96.0 192.0 384.0
Dose
24.00 48.00 96.00 192.00 384.00
MEL | log(Fa/Fu)
log(Fa/Fu)
0.0
-0.6
-1.2
1.4 1.7 2.0 2.3 2.6
log(D)
1.38 1.68 1.98 2.28 2.58
CBD | Fa
Fa
1.0
0.5
0.0
10.0 20.0 40.0 80.0 160.0
Dose
10.00 20.00 40.00 80.00 160.00
CBD | log(Fa/Fu)
log(Fa/Fu)
1.0
-0.3
-1.5
1.0 1.3 1.6 1.9 2.2
log(D)
1.00 1.30 1.60 1.90 2.20
THC | Fa
Fa
1.0
0.5
0.0
7.5 15.0 30.0 60.0 120.0
Dose
7.50 15.00 30.00 60.00 120.00
THC | log(Fa/Fu)
log(Fa/Fu)
1.0
-0.3
-1.5
0.9 1.2 1.5 1.8 2.1
log(D)
0.88 1.18 1.48 1.78 2.08
CP | Fa
Fa
0.6
0.3
0.0
5.0 10.0 20.0 40.0 80.0
Dose
5.00 10.00 20.00 40.00 80.00
CP | log(Fa/Fu)
log(Fa/Fu)
0.2
-0.5
-1.2
0.7 1.0 1.3 1.6 1.9
log(D)
0.70 1.00 1.30 1.60 1.90
CBD+MEL | Fa
Fa
1.0
0.5
0.0
15.0 30.0 60.0 120.0 240.0
Dose
15.00 30.00 60.00 120.00 240.00
CBD+MEL | log(Fa/Fu)
log(Fa/Fu)
1.0
-0.3
-1.5
1.2 1.5 1.8 2.1 2.4
log(D)
1.18 1.48 1.78 2.08 2.38
CBD+MEL | CI
CI
2.0
1.0
0.0
0.1 0.1 0.5 0.8 0.8
Fa
0.09 0.14 0.53 0.81 0.82
CBD+MEL | log(CI)
log(CI)
0.3
0.1
-0.2
0.1 0.1 0.5 0.8 0.8
Fa
0.09 0.14 0.53 0.81 0.82
CBD+MEL | DRI CBD
DRI
80.0
40.0
0.0
0.1 0.1 0.5 0.8 0.8
Fa
CBD+MEL | DRI CBD
CBD+MEL | DRI MEL
CBD+MEL | logDRI CBD
log(DRI)
2.0
0.8
-0.5
0.1 0.1 0.5 0.8 0.8
Fa
CBD+MEL | logDRI CBD
CBD+MEL | logDRI MEL
THC+MEL | Fa
Fa
1.0
0.5
0.0
29.0 58.0 116.0 232.0 464.0
Dose
29.00 58.00 116.00 232.00 464.00
THC+MEL | log(Fa/Fu)
log(Fa/Fu)
1.0
-0.3
-1.5
1.5 1.8 2.1 2.4 2.7
log(D)
1.46 1.76 2.06 2.37 2.67

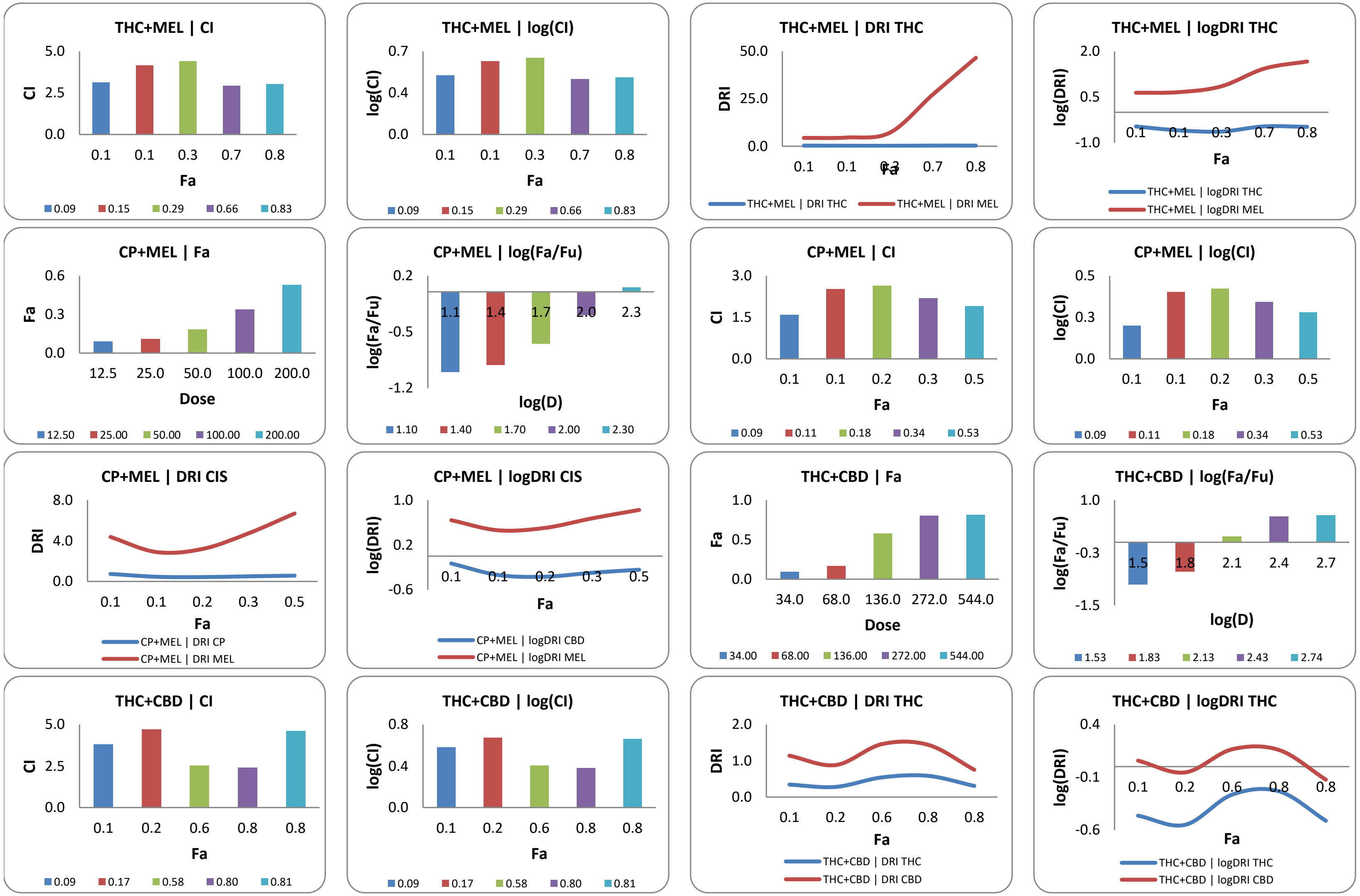

THC+MEL | CI
CI
Fa
0.09 0.15 0.29 0.66 0.83
THC+MEL | log(CI)
log(CI)
Fa
0.09 0.15 0.29 0.66 0.83
THC+MEL | DRI THC
DRI
Fa
THC+MEL | DRI THC
THC+MEL | DRI MEL
THC+MEL | logDRI THC
log(DRI)
Fa
THC+MEL | logDRI THC
THC+MEL | logDRI MEL
CP+MEL | Fa
Fa
Dose
12.50 25.00 50.00 100.00 200.00
CP+MEL | log(Fa/Fu)
log(Fa/Fu)
log(D)
1.10 1.40 1.70 2.00 2.30
CP+MEL | CI
CI
Fa
0.09 0.11 0.18 0.34 0.53
CP+MEL | log(CI)
log(CI)
Fa
0.09 0.11 0.18 0.34 0.53
CP+MEL | DRI CIS
DRI
Fa
CP+MEL | DRI CP
CP+MEL | DRI MEL
CP+MEL | logDRI CIS
log(DRI)
Fa
CP+MEL | logDRI CBD
CP+MEL | logDRI MEL
THC+CBD | Fa
Fa
Dose
34.00 68.00 136.00 272.00 544.00
THC+CBD | log(Fa/Fu)
log(Fa/Fu)
log(D)
1.53 1.83 2.13 2.43 2.74
THC+CBD | CI
CI
Fa
0.09 0.17 0.58 0.80 0.81
THC+CBD | log(CI)
log(CI)
Fa
0.09 0.17 0.58 0.80 0.81
THC+CBD | DRI THC
DRI
Fa
THC+CBD | DRI THC
THC+CBD | DRI CBD
THC+CBD | logDRI THC
log(DRI)
Fa
THC+CBD | logDRI THC
THC+CBD | logDRI CBD

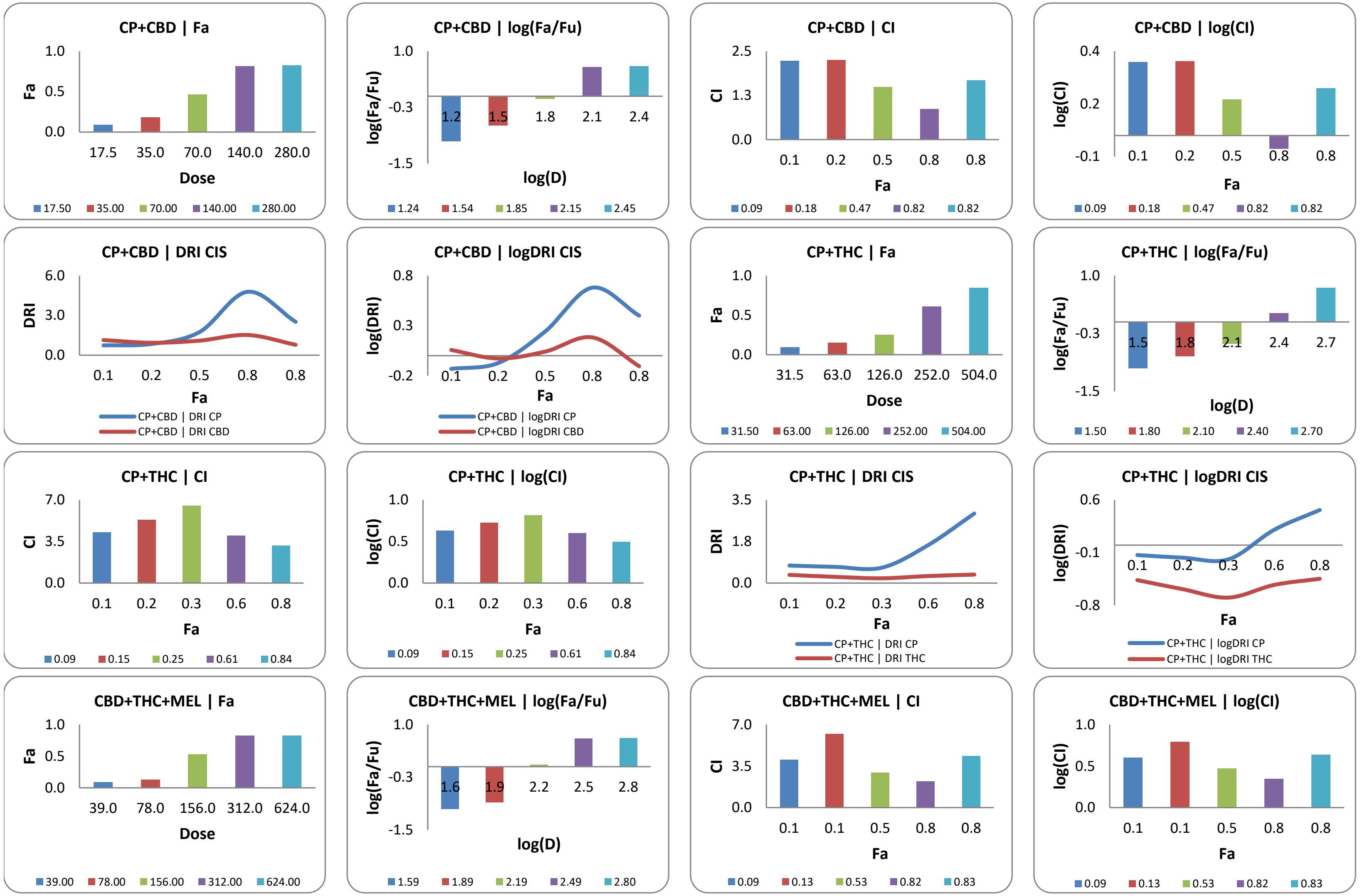
CP+CBD | Fa
Fa
1.0
0.5
0.0
17.5 35.0 70.0 140.0 280.0
Dose
17.50 35.00 70.00 140.00 280.00
CP+CBD | log(Fa/Fu)
log(Fa/Fu)
1.0
-0.3
-1.5
1.2 1.5 1.8 2.1 2.4
log(D)
1.24 1.54 1.85 2.15 2.45
CP+CBD | CI
CI
2.5
1.3
0.0
0.1 0.2 0.5 0.8 0.8
Fa
0.09 0.18 0.47 0.82 0.82
CP+CBD | log(CI)
log(CI)
0.4
0.2
-0.1
0.1 0.2 0.5 0.8 0.8
Fa
0.09 0.18 0.47 0.82 0.82
CP+CBD | DRI CIS
DRI
6.0
3.0
0.0
0.1 0.2 0.5 0.8 0.8
Fa
CP+CBD | DRI CP
CP+CBD | DRI CBD
CP+CBD | logDRI CIS
log(DRI)
0.8
0.3
-0.2
0.1 0.2 0.5 0.8 0.8
Fa
CP+CBD | logDRI CP
CP+CBD | logDRI CBD
CP+THC | Fa
Fa
1.0
0.5
0.0
31.5 63.0 126.0 252.0 504.0
Dose
31.50 63.00 126.00 252.00 504.00
CP+THC | log(Fa/Fu)
log(Fa/Fu)
1.0
-0.3
-1.5
1.5 1.8 2.1 2.4 2.7
log(D)
1.50 1.80 2.10 2.40 2.70
CP+THC | CI
CI
7.0
3.5
0.0
0.1 0.2 0.3 0.6 0.8
Fa
0.09 0.15 0.25 0.61 0.84
CP+THC | log(CI)
log(CI)
1.0
0.5
0.0
0.1 0.2 0.3 0.6 0.8
Fa
0.09 0.15 0.25 0.61 0.84
CP+THC | DRI CIS
DRI
3.5
1.8
0.0
0.1 0.2 0.3 0.6 0.8
Fa
CP+THC | DRI CP
CP+THC | DRI THC
CP+THC | logDRI CIS
log(DRI)
0.6
-0.1
-0.8
0.1 0.2 0.3 0.6 0.8
Fa
CP+THC | logDRI CP
CP+THC | logDRI THC
CBD+THC+MEL | Fa
Fa
1.0
0.5
0.0
39.0 78.0 156.0 312.0 624.0
Dose
39.00 78.00 156.00 312.00 624.00
CBD+THC+MEL | log(Fa/Fu)
log(Fa/Fu)
1.0
-0.3
-1.5
1.6 1.9 2.2 2.5 2.8
log(D)
1.59 1.89 2.19 2.49 2.80
CBD+THC+MEL | CI
CI
7.0
3.5
0.0
0.1 0.1 0.5 0.8 0.8
Fa
0.09 0.13 0.53 0.82 0.83
CBD+THC+MEL | log(CI)
log(CI)
1.0
0.5
0.0
0.1 0.1 0.5 0.8 0.8
Fa
0.09 0.13 0.53 0.82 0.83

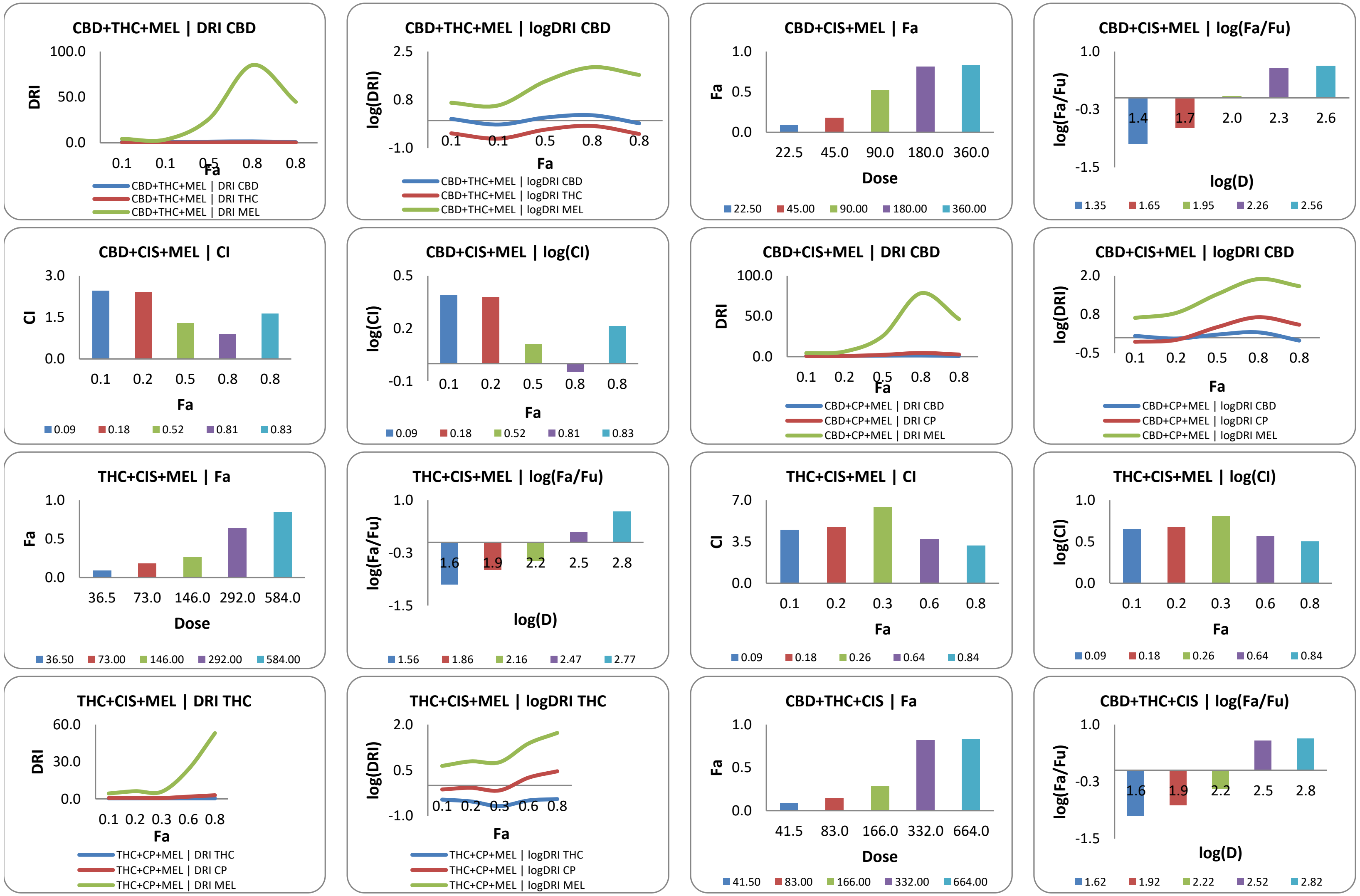
CBD+THC+MEL | DRI CBD
DRI
100.0
50.0
0.0
0.1 0.1 0.5 0.8 0.8
Fa
CBD+THC+MEL | DRI CBD
CBD+THC+MEL | DRI THC
CBD+THC+MEL | DRI MEL
CBD+THC+MEL | logDRI CBD
log(DRI)
2.5
0.8
-1.0
0.1 0.1 0.5 0.8 0.8
Fa
CBD+THC+MEL | logDRI CBD
CBD+THC+MEL | logDRI THC
CBD+THC+MEL | logDRI MEL
CBD+CIS+MEL | Fa
Fa
1.0
0.5
0.0
22.5 45.0 90.0 180.0 360.0
Dose
22.50 45.00 90.00 180.00 360.00
CBD+CIS+MEL | log(Fa/Fu)
log(Fa/Fu)
1.0
-0.3
-1.5
1.4 1.7 2.0 2.3 2.6
log(D)
1.35 1.65 1.95 2.26 2.56
CBD+CIS+MEL | CI
CI
3.0
1.5
0.0
0.1 0.2 0.5 0.8 0.8
Fa
0.09 0.18 0.52 0.81 0.83
CBD+CIS+MEL | log(CI)
log(CI)
0.5
0.2
-0.1
0.1 0.2 0.5 0.8 0.8
Fa
0.09 0.18 0.52 0.81 0.83
CBD+CIS+MEL | DRI CBD
DRI
100.0
50.0
0.0
0.1 0.2 0.5 0.8 0.8
Fa
CBD+CP+MEL | DRI CBD
CBD+CP+MEL | DRI CP
CBD+CP+MEL | DRI MEL
CBD+CIS+MEL | logDRI CBD
log(DRI)
2.0
0.8
-0.5
0.1 0.2 0.5 0.8 0.8
Fa
CBD+CP+MEL | logDRI CBD
CBD+CP+MEL | logDRI CP
CBD+CP+MEL | logDRI MEL
THC+CIS+MEL | Fa
Fa
1.0
0.5
0.0
36.5 73.0 146.0 292.0 584.0
Dose
36.50 73.00 146.00 292.00 584.00
THC+CIS+MEL | log(Fa/Fu)
log(Fa/Fu)
1.0
-0.3
-1.5
1.6 1.9 2.2 2.5 2.8
log(D)
1.56 1.86 2.16 2.47 2.77
THC+CIS+MEL | CI
CI
7.0
3.5
0.0
0.1 0.2 0.3 0.6 0.8
Fa
0.09 0.18 0.26 0.64 0.84
THC+CIS+MEL | log(CI)
log(CI)
1.0
0.5
0.0
0.1 0.2 0.3 0.6 0.8
Fa
0.09 0.18 0.26 0.64 0.84
THC+CIS+MEL | DRI THC
DRI
60.0
30.0
0.0
0.1 0.2 0.3 0.6 0.8
Fa
THC+CP+MEL | DRI THC
THC+CP+MEL | DRI CP
THC+CP+MEL | DRI MEL
THC+CIS+MEL | logDRI THC
log(DRI)
2.0
0.5
-1.0
0.1 0.2 0.3 0.6 0.8
Fa
THC+CP+MEL | logDRI THC
THC+CP+MEL | logDRI CP
THC+CP+MEL | logDRI MEL
CBD+THC+CIS | Fa
Fa
1.0
0.5
0.0
41.5 83.0 166.0 332.0 664.0
Dose
41.50 83.00 166.00 332.00 664.00
CBD+THC+CIS | log(Fa/Fu)
log(Fa/Fu)
1.0
-0.3
-1.5
1.6 1.9 2.2 2.5 2.8
log(D)
1.62 1.92 2.22 2.52 2.82

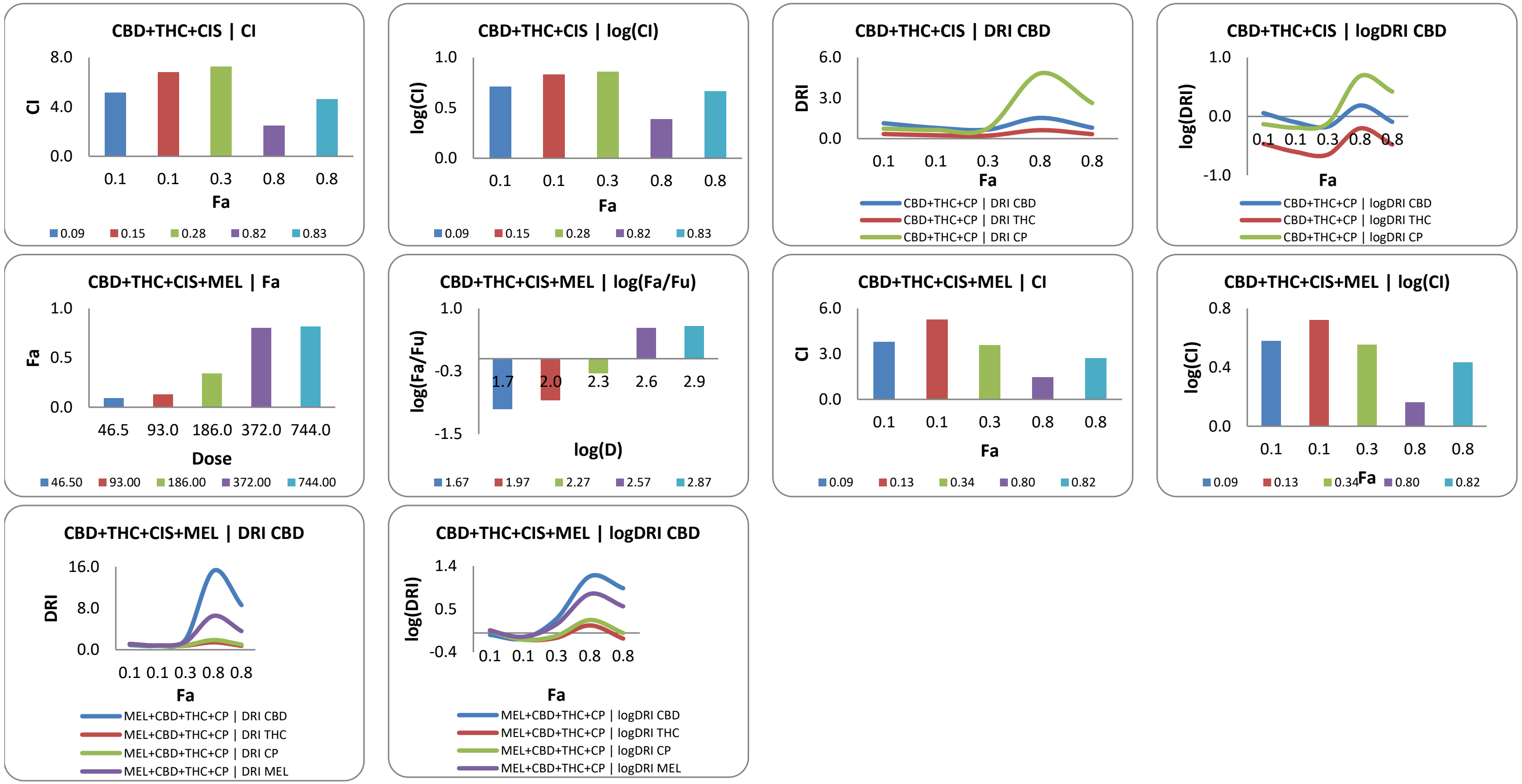
CBD+THC+CIS | CI
CI
Fa
0.09 0.15 0.28 0.82 0.83
CBD+THC+CIS | log(CI)
log(CI)
Fa
CBD+THC+CIS | DRI CBD
DRI
Fa
CBD+THC+CP | DRI CBD
CBD+THC+CP | DRI THC
CBD+THC+CP | DRI CP
CBD+THC+CIS | logDRI CBD
log(DRI)
Fa
CBD+THC+CP | logDRI CBD
CBD+THC+CP | logDRI THC
CBD+THC+CP | logDRI CP
CBD+THC+CIS+MEL | Fa
Fa
Dose
46.50 93.00 186.00 372.00 744.00
CBD+THC+CIS+MEL | log(Fa/Fu)
log(Fa/Fu)
log(D)
1.67 1.97 2.27 2.57 2.87
CBD+THC+CIS+MEL | CI
CI
Fa
0.09 0.13 0.34 0.80 0.82
CBD+THC+CIS+MEL | log(CI)
log(CI)
Fa
CBD+THC+CIS+MEL | DRI CBD
DRI
Fa
MEL+CBD+THC+CP | DRI CBD
MEL+CBD+THC+CP | DRI THC
MEL+CBD+THC+CP | DRI CP
MEL+CBD+THC+CP | DRI MEL
CBD+THC+CIS+MEL | logDRI CBD
log(DRI)
Fa
MEL+CBD+THC+CP | logDRI CBD
MEL+CBD+THC+CP | logDRI THC
MEL+CBD+THC+CP | logDRI CP
MEL+CBD+THC+CP | logDRI MEL
