# Supplementary material for: A selective low-dose cisplatin strategy enabled by melatonin and cannabinoids for targeting osteosarcoma and chondrosarcoma
Source: Naunyn Schmiedebergs Arch Pharmacol. 2026 Jun 27;399(12):19709–22. doi: 10.1007/s00210-026-05551-y (PMC13593660; doi:10.1007/s00210-026-05551-y)
Supplement: Supplementary file 5 [file 210_2026_5551_MOESM1_ESM.docx]

**Supplemental Material**

| **Supplemental Table 1.** Description of synergism or antagonism in drug combination studies analyzed with the Combination index (CI) method. | | | |
| --- | --- | --- | --- |
| Range of CI | Description | Range of CI | Description |
| < 0.1 | Very strong synergy | 1.10 - 1.20 | Mild antagonism |
| 0.1 - 0.3 | Strong synergy | 1.20 - 1.45 | Moderate antagonism |
| 0.3 - 0.7 | Synergy | 1.45 - 3.3 | Antagonism |
| 0.7 - 0.85 | Moderate synergy | 3.3 - 10 | Strong antagonism |
| 0.85 - 0.90 | Light synergy | 10 > | Very strong antagonism |
| 0.90 - 1.10 | Additive |  |  |

| **Supplemental Table 2.** D*m*/ED50 (μM) and Combination Index (*CI*) values for the molecules, single dose or in combination, in MG63 cells. | | | | | | | | | | | | | | |
| --- | --- | --- | --- | --- | --- | --- | --- | --- | --- | --- | --- | --- | --- | --- |
| Combination Index (*CI*) Values at | | | | | | | | | | | | | | |
| **MG63** | ***m*** | **D*m*** | ***r*** |  | ***m*** | **D*m*** | ***r*** | ***CI*** |  | ***m*** | **D*m*** | ***r*** | ***CI*** |  |
| **MEL** | 0.83 | 443.83 | 0.98 | **CBD+MEL** | 1.95 | 49.44 | 0.97 | 1.16 | **CBD+THC+MEL** | 2.01 | 44.43 | 0.93 | 3.51 |  |
| **CBD** | 1.83 | 30.66 | 0.98 | **THC+MEL** | 1.81 | 66.70 | 0.96 | 4.23 | **CBD+CP+MEL** | 1.91 | 53.85 | 0.95 | 1.90 |  |
| **THC** | 1.97 | 29.91 | 0.96 | **CP+MEL** | 1.48 | 167.46 | 0.95 | 2.15 | **THC+CP+MEL** | 1.78 | 70.95 | 0.991 | 4.71 |  |
| **CP** | 1.47 | 25.09 | 0.98 | **THC+CBD** | 1.99 | 46.15 | 0.95 | 3.61 | **CBD+THC+CP** | 1.92 | 52.36 | 0.97 | 4.53 |  |
|  |  |  |  | **CP+CBD** | 1.97 | 47.93 | 0.96 | 1.89 | **MEL+CBD+THC+CP** | 1.90 | 148.40 | 0.96 | 2.86 |  |
|  |  |  |  | **CP+THC** | 1.94 | 50.64 | 0.96 | 4.87 |  |  |  |  |  |  |

| **Supplemental Table 3.** D*m* (IC50 or ED50) and Combination Index (*CI*) values for the molecules, single dose or in combination, in Saos2 cells. 0<*CI*<1, *CI*=1, *CI*>1 indicate synergism, additive and antagonism, respectively. 'D*m*' (μM): Inhibitor concentration (IC50) or Median Effective Dose 50 (ED50). | | | | | | | | | | | | | | |
| --- | --- | --- | --- | --- | --- | --- | --- | --- | --- | --- | --- | --- | --- | --- |
| Combination Index (*CI*) Values at | | | | | | | | | | | | | | |
| **Saos2** | ***m*** | **D*m*** | ***r*** |  | ***m*** | **D*m*** | ***r*** | ***Cl*** |  | ***m*** | **D*m*** | ***r*** | ***CI*** |  |
| **MEL** | 1.29 | 131.21 | 0.99 | **CBD+MEL** | 1.32 | 65.80 | 0.95 | 1.18 | **CBD+THC+MEL** | 1.27 | 78.24 | 0.94 | 3.32 |  |
| **CBD** | 1.30 | 46.22 | 0.97 | **THC+MEL** | 1.27 | 76.82 | 0.99 | 3.57 | **CBD+CP+MEL** | 1.30 | 69.15 | 0.96 | 2.38 |  |
| **THC** | 1.33 | 50.57 | 0.98 | **CP+MEL** | 1.39 | 53.49 | 0.99 | 1.01 | **THC+CP+MEL** | 2.13 | 13.34 | 0.99 | 0.76 |  |
| **CP** | 1.09 | 45.29 | 0.97 | **THC+CBD** | 1.29 | 72.67 | 0.93 | 3.42 | **CBD+THC+CP** | 1.39 | 53.31 | 0.98 | 4.26 |  |
|  |  |  |  | **CP+CBD** | 1.27 | 78.80 | 0.93 | 1.72 | **MEL+CBD+THC+CP** | 1.46 | 235.52 | 0.97 | 3.62 |  |
|  |  |  |  | **CP+THC** | 1.28 | 74.68 | 0.98 | 3.94 |  |  |  |  |  |  |

| **Supplemental Table 4.** D*m* (IC50 or ED50) and Combination Index (*CI*) values for the molecules, single dose or in combination, in SW1353 cells. 0<*CI*<1, *CI*=1, *CI*>1 indicate synergism, additive and antagonism, respectively. 'D*m*' (μM): Inhibitor concentration (IC50) or Median Effective Dose 50 (ED50). | | | | | | | | | | | | | |
| --- | --- | --- | --- | --- | --- | --- | --- | --- | --- | --- | --- | --- | --- |
| Combination Index (*CI*) Values at | | | | | | | | | | | | | |
| **SW1353** | ***m*** | **D*m*** | ***r*** |  | ***m*** | **D*m*** | ***r*** | ***CI*** |  | ***m*** | **D*m*** | ***r*** | ***CI*** |
| **MEL** | 0.76 | 455.85 | 0.98 | **CBD+MEL** | 1.57 | 69.09 | 0.96 | 1.11 | **CBD+THC+MEL** | 1.62 | 60.68 | 0.96 | 3.96 |
| **CBD** | 1.60 | 47.87 | 0.95 | **THC+MEL** | 1.47 | 90.87 | 0.99 | 3.54 | **CBD+CP+MEL** | 1.55 | 72.08 | 0.98 | 1.74 |
| **THC** | 1.42 | 41.57 | 0.98 | **CP+MEL** | 1.70 | 49.10 | 0.96 | 0.90 | **THC+CP+MEL** | 1.45 | 96.44 | 0.98 | 4.50 |
| **CP** | 0.96 | 60.45 | 1.00 | **THC+CBD** | 1.53 | 77.46 | 0.96 | 3.61 | **CBD+THC+CP** | 1.60 | 63.75 | 0.96 | 5.26 |
|  |  |  |  | **CP+CBD** | 1.54 | 73.39 | 0.97 | 1.70 | **MEL+CBD+THC+CP** | 1.58 | 238.60 | 0.96 | 3.36 |
|  |  |  |  | **CP+THC** | 1.46 | 92.70 | 0.98 | 4.66 |  |  |  |  |  |

| **Supplemental Table 5.** D*m* (IC50 or ED50) and Combination Index (*CI*) values for the molecules, single dose or in combination, in FL cells. 0<*CI*<1, *CI*=1, *CI*>1 indicate synergism, additive and antagonism, respectively. 'D*m*' (μM): Inhibitor concentration (IC50) or Median Effective Dose 50 (ED50). | | | | | | | | | | | | | |
| --- | --- | --- | --- | --- | --- | --- | --- | --- | --- | --- | --- | --- | --- |
| Combination Index (*CI*) Values at | | | | | | | | | | | | | |
| **FL** | ***m*** | **D*m*** | ***r*** |  | ***m*** | **D*m*** | ***r*** | ***CI*** |  | ***m*** | **D*m*** | ***r*** | ***CI*** |
| **MEL** | 0.65 | 848.26 | 0.99 | **CBD+MEL** | 1.53 | 56.21 | 0.97 | 1.00 | **CBD+THC+MEL** | 1.59 | 48.69 | 0.93 | 3.42 |
| **CBD** | 1.53 | 41.04 | 0.96 | **THC+MEL** | 1.58 | 49.87 | 0.97 | 3.32 | **CBD+CP+MEL** | 1.58 | 49.44 | 0.95 | 1.59 |
| **THC** | 1.62 | 38.65 | 0.96 | **CP+MEL** | 1.65 | 42.00 | 0.98 | 0.96 | **THC+CP+MEL** | 1.58 | 49.96 | 0.98 | 3.51 |
| **CP** | 1.16 | 44.26 | 0.97 | **THC+CBD** | 1.60 | 47.61 | 0.93 | 3.26 | **CBD+THC+CP** | 1.55 | 54.11 | 0.95 | 4.15 |
|  |  |  |  | **CP+CBD** | 1.62 | 44.94 | 0.95 | 1.67 | **MEL+CBD+THC+CP** | 1.54 | 162.82 | 0.95 | 2.32 |
|  |  |  |  | **CP+THC** | 1.69 | 38.53 | 0.96 | 3.91 |  |  |  |  |  |

| **Supplemental Table 6.** Dose-Reduction Index (DRI) for each drug in the combination (Fa: 0.50). | | | | | | | | | |
| --- | --- | --- | --- | --- | --- | --- | --- | --- | --- |
| **MG63** | **Drug alone** | | | |  | **Dose Reduction Index (DRI)** | | | |
| **CBD+MEL** | 30.66 | 443.83 |  |  |  | 0.97 | 80.92 |  |  |
| **THC+MEL** | 29.91 | 443.83 |  |  |  | 0.26 | 32.14 |  |  |
| **CP+MEL** | 25.09 | 443.83 |  |  |  | 0.54 | 16.58 |  |  |
| **THC+CBD** | 29.91 | 30.66 |  |  |  | 0.42 | 1.09 |  |  |
| **CP+CBD** | 25.09 | 30.66 |  |  |  | 1.38 | 1.09 |  |  |
| **CP+THC** | 25.09 | 29.91 |  |  |  | 0.87 | 0.30 |  |  |
| **CBD+THC+MEL** | 30.66 | 29.91 | 443.83 |  |  | 1.19 | 0.46 | 117.69 |  |
| **CBD+CP+MEL** | 30.66 | 25.09 | 443.83 |  |  | 1.12 | 1.42 | 96.93 |  |
| **THC+CP+MEL** | 29.91 | 25.09 | 443.83 |  |  | 0.30 | 0.83 | 40.62 |  |
| **CBD+THC+CP** | 30.66 | 29.91 | 25.09 |  |  | 1.03 | 0.40 | 1.28 |  |
| **MEL+CBD+THC+CP** | 443.83 | 30.66 | 29.91 | 25.09 |  | 16.16 | 1.00 | 1.27 | 1.85 |

| **Supplemental Table 7.** Dose-Reduction Index (DRI) for each drug in the combination. DRI = 1, >1, and <1 indicate no dose reduction, appropriate dose reduction, and inappropriate dose reduction for each drug in the combination, respectively. | | | | | | | | | | |
| --- | --- | --- | --- | --- | --- | --- | --- | --- | --- | --- |
| **Saos2** | **Drug alone** | | | |  | **Dose Reduction Index (DRI)** | | | |  |
| **CBD+MEL** | 46.22 | 131.21 |  |  |  | 1.11 | 6.33 |  |  |  |
| **THC+MEL** | 50.57 | 131.21 |  |  |  | 0.31 | 3.79 |  |  |  |
| **CP+MEL** | 45.29 | 131.21 |  |  |  | 0.89 | 4.26 |  |  |  |
| **THC+CBD** | 50.57 | 46.22 |  |  |  | 0.49 | 1.07 |  |  |  |
| **CP+CBD** | 45.29 | 46.22 |  |  |  | 1.63 | 1.21 |  |  |  |
| **CP+THC** | 45.29 | 50.57 |  |  |  | 0.98 | 0.36 |  |  |  |
| **CBD+THC+MEL** | 46.22 | 50.57 | 131.21 |  |  | 1.13 | 0.52 | 6.44 |  |  |
| **CBD+CP+MEL** | 46.22 | 45.29 | 131.21 |  |  | 0.91 | 1.16 | 5.13 |  |  |
| **THC+CP+MEL** | 50.57 | 45.29 | 131.21 |  |  | 0.23 | 0.52 | 2.77 |  |  |
| **CBD+THC+CP** | 46.22 | 50.57 | 45.29 |  |  | 1.00 | 0.46 | 1.32 |  |  |
| **MEL+CBD+THC+CP** | 131.21 | 46.22 | 50.57 | 45.29 |  | 1.09 | 0.92 | 1.34 | 1.82 |  |

| **Supplemental Table 8.** Dose-Reduction Index (DRI) for each drug in the combination. DRI = 1, >1, and <1 indicate no dose reduction, appropriate dose reduction, and inappropriate dose reduction for each drug in the combination, respectively. | | | | | | | | | |
| --- | --- | --- | --- | --- | --- | --- | --- | --- | --- |
| **SW1353** | **Drug alone** | | | |  | **Dose Reduction Index (DRI)** | | | |
| **CBD+MEL** | 47.87 | 455.85 |  |  |  | 1.08 | 30.22 |  |  |
| **THC+MEL** | 41.57 | 455.85 |  |  |  | 0.30 | 18.01 |  |  |
| **CP+MEL** | 60.45 | 455.85 |  |  |  | 0.54 | 4.38 |  |  |
| **THC+CBD** | 41.57 | 47.87 |  |  |  | 0.41 | 1.13 |  |  |
| **CP+CBD** | 60.45 | 47.87 |  |  |  | 2.12 | 1.10 |  |  |
| **CP+THC** | 60.45 | 41.57 |  |  |  | 1.32 | 0.29 |  |  |
| **CBD+THC+MEL** | 47.87 | 41.57 | 47.87 |  |  | 1.09 | 0.40 | 32.80 | 1.09 |
| **CBD+CP+MEL** | 47.87 | 60.45 | 47.87 |  |  | 1.13 | 2.20 | 32.21 | 1.13 |
| **THC+CP+MEL** | 41.57 | 60.45 | 41.57 |  |  | 0.31 | 1.40 | 18.67 | 0.31 |
| **CBD+THC+CP** | 47.87 | 41.57 | 47.87 |  |  | 0.99 | 0.36 | 1.92 | 0.99 |
| **MEL+CBD+THC+CP** | 455.85 | 47.87 | 41.57 | 60.45 |  | 5.50 | 0.97 | 1.11 | 2.72 |

| **Supplemental Table 9.** Dose-Reduction Index (DRI) for each drug in the combination. DRI = 1, >1, and <1 indicate no dose reduction, appropriate dose reduction, and inappropriate dose reduction for each drug in the combination, respectively. | | | | | | | | | |
| --- | --- | --- | --- | --- | --- | --- | --- | --- | --- |
| **FL** | **Drug alone** | | | |  | **Dose Reduction Index (DRI)** | | | |
| **CBD+MEL** | 41.04 | 848.26 |  |  |  | 1.13 | 104.22 |  |  |
| **THC+MEL** | 38.65 | 848.26 |  |  |  | 0.33 | 67.79 |  |  |
| **CP+MEL** | 44.26 | 848.26 |  |  |  | 0.48 | 7.06 |  |  |
| **THC+CBD** | 38.65 | 41.04 |  |  |  | 0.49 | 1.26 |  |  |
| **CP+CBD** | 44.26 | 41.04 |  |  |  | 1.96 | 1.19 |  |  |
| **CP+THC** | 44.26 | 38.65 |  |  |  | 1.48 | 0.37 |  |  |
| **CBD+THC+MEL** | 41.04 | 38.65 | 41.04 |  |  | 1.25 | 0.48 | 139.20 |  |
| **CBD+CP+MEL** | 41.04 | 44.26 | 41.04 |  |  | 1.27 | 2.11 | 139.45 |  |
| **THC+CP+MEL** | 38.65 | 44.26 | 38.65 |  |  | 0.39 | 1.54 | 91.93 |  |
| **CBD+THC+CP** | 41.04 | 38.65 | 41.04 |  |  | 1.15 | 0.45 | 1.85 |  |
| **MEL+CBD+THC+CP** | 848.26 | 41.04 | 38.65 | 44.26 |  | 26.25 | 1.24 | 1.54 | 3.04 |

| **Supplemental Table 10.** Primers used for qRT-PCR analysis. | | |
| --- | --- | --- |
| **Gene** | **Forward (5′→3′)** | **Reverse (5′→3′)** |
| ***CASP3*** | GGAAGCGAATCAATGGACTCTGG | GCATCGACATCTGTACCAGACC |
| ***CASP8*** | AGAAGAGGGTCATCCTGGGAGA | TCAGGACTTCCTTCAAGGCTGC |
| ***CASP9*** | GTTTGAGGACCTTCGACCAGCT | CAACGTACCAGGAGCCACTCTT |
| ***BAX*** | TCAGGATGCGTCCACCAAGAAG | TGTGTCCACGGCGGCAATCATC |
| ***BCL2*** | ATCGCCCTGTGGATGACTGAGT | GCCAGGAGAAATCAAACAGAGGC |
| ***GAPDH*** | GTCTCCTCTGACTTCAACAGCG | ACCACCCTGTTGCTGTAGCCAA |

**Supplemental Figure 1.** Conservative isobolograms of binary combinations of molecules (μM) in MG63 cells. The drawn line indicates additivity; those below are considered synergistic, and those above are considered antagonistic.

**Supplemental Figure 2.** Conservative isobolograms of binary combinations of molecules (μM) in Saos2 cells. The drawn line indicates additivity; those below are considered synergistic, and those above are considered antagonistic. The units on the x- and y-axes are the doses of the drugs used.

**Supplemental Figure 3.** Conservative isobolograms of binary combinations of molecules (μM) in SW1353 cells. The drawn line indicates additivity; those below are considered synergistic, and those above are considered antagonistic. The units on the x- and y-axes are the doses of the drugs used.

**Supplemental Figure 4.** Conservative isobolograms of binary combinations of molecules (μM) in FL cells. The drawn line indicates additivity; those below are considered synergistic, and those above are considered antagonistic. The units on the x- and y-axes are the doses of the drugs used.

| **Supplemental Table 11.** % Cytotoxicity values of test substances at D*m* concentrations. * | | | | | |
| --- | --- | --- | --- | --- | --- |
| % Cytotoxicity | SW1353 | Saos2 | MG63 | FL | TSI |
| MEL | 20.71 | 29.44 | 23.13 | 15.50 | 1.57 |
| CBD | 27.14 | 15.56 | 26.25 | 20.00 | 1.14 |
| THC | 10.00 | 9.44 | 14.38 | 9.45 | 1.19 |
| CP | 10.00 | 13.89 | 14.38 | 12.50 | 1.02 |
| CBD+MEL | 27.86 | 14.44 | 25.00 | 13.50 | 1.66 |
| THC+MEL | 5.00 | 4.44 | 9.38 | 4.57 | 1.37 |
| CP+MEL | 7.43 | 7.30 | 8.35 | 6.50 | 1.18 |
| THC+CBD | 29.43 | 28.11 | 26.25 | 21.50 | 1.29 |
| CP+CBD | 20.71 | 22.00 | 29.75 | 19.50 | 1.23 |
| CP+THC | 17.14 | 19.78 | 25.00 | 13.50 | 1.52 |
| THC+CBD+MEL | 35.71 | 32.56 | 38.75 | 28.53 | 1.25 |
| CP+CBD+MEL | 39.29 | 28.33 | 32.13 | 30.22 | 1.10 |
| CP+THC+MEL | 17.86 | 16.11 | 18.13 | 15.50 | 1.12 |
| CP+THC+CBD | 17.14 | 16.67 | 75.00 | 35.50 | 1.02 |
| MEL+CBD+THC+CP | 45.00 | 44.44 | 41.25 | 37.62 | 1.15 |

*Values are calculated assuming that the damage caused by Triton x-100 to the membrane is 100%.

| **Supplemental Table 12.** Expression levels of 5 genes in cells treated with MEL, CBD+MEL, THC+MEL, and CP+MEL. | | | | | | | | | |
| --- | --- | --- | --- | --- | --- | --- | --- | --- | --- |
| **MG63** | MEL | | CBD+MEL | | THC+MEL | | CP+MEL | |  |
| Gene | FR* | *p*-Value | FR | *p*-Value | FR | *p*-Value | FR | *p*-Value |  |
| GAPDH | 1.00 | non | 1.00 | non | 1.00 | non | 1.00 | non |  |
| Caspase-3 | 11.63 | 0.041 | 4.21 | 0.000 | 30.38 | 0.004 | 5.06 | 0.001 |  |
| Caspase-8 | 23.18 | 0.005 | 10.78 | 0.000 | 14.83 | 0.000 | 7.89 | 0.000 |  |
| Caspase-9 | 48.50 | 0.039 | 5.88 | 0.027 | 39.95 | 0.000 | 10.37 | 0.000 |  |
| Bax | 14.07 | 0.014 | 26.72 | 0.000 | 98.02 | 0.002 | 10.89 | 0.004 |  |
| Bcl-2 | 4.20 | 0.003 | -1.49 | 0.030 | 3.46 | 0.015 | 5.74 | 0.000 |  |
| **HC** | MEL | | CBD+MEL | | THC+MEL | | CP+MEL | |  |
| Gene | FR | *p*-Value | FR | *p*-Value | FR | *p*-Value | FR | *p*-Value |  |
| GAPDH | 1.00 | non | 1.00 | non | 1.00 | non | 1.00 | non |  |
| Caspase-3 | -1.55 | 0.027 | -5.01 | 0.000 | -1.95 | 0.002 | -3.66 | 0.000 |  |
| Caspase-8 | -3.47 | 0.013 | 1.83 | 0.022 | 3.28 | 0.036 | -1.14 | 0.047 |  |
| Caspase-9 | -2.42 | 0.018 | 1.78 | 0.012 | -1.20 | 0.045 | -3.51 | 0.036 |  |
| Bax | 1.74 | 0.034 | 2.79 | 0.005 | 5.03 | 0.014 | 1.62 | 0.045 |  |
| Bcl-2 | 1.48 | 0.041 | -2.72 | 0.048 | 1.04 | 0.046 | -2.40 | 0.030 |  |

*FR: Fold Regulation

**Supplemental Figure 5.** DAPI/Rhodamine 123 staining in Saos2 cancer cell lines. All scales are 100 µm.

**Supplemental Figure 6.** DAPI/Rhodamine 123 staining in SW1353 cancer cell lines. All scales are 100 µm.

**Supplemental Figure 7.** DAPI/Rhodamine 123 staining in FL normal cell lines. All scales are 100 µm.

| **Supplemental Table 13.** Results of migration % area analysis of the cells using ImageR software. | | | | | | |
| --- | --- | --- | --- | --- | --- | --- |
|  | **FL** | | | **MG63** | | |
| **% Area** | **Day 1** | **Day 3** | **% Gap** | **Day 1** | **Day 3** | **% Gap** |
| MEL | 94.73 | 61.77 | 32.96 | 57.47 | 70.78 | -13.31 |
| CBD | 57.33 | 56.94 | 0.39 | 55.78 | 50.82 | 4.96 |
| THC | 87.65 | 64.04 | 23.61 | 47.69 | 48.48 | -0.79 |
| CP | 57.89 | 54.85 | 3.04 | 39.33 | 23.36 | 15.97 |
| CBD+MEL | 66.72 | 67.67 | -0.95 | 69.04 | 56.18 | 12.86 |
| THC+MEL | 80.38 | 63.91 | 16.47 | 34.44 | 49.34 | -14.9 |
| CP+MEL | 51.64 | 60.35 | -8.71 | 71.70 | 71.97 | -0.27 |
| THC+CBD | 54.18 | 49.48 | 4.7 | 49.88 | 66.77 | -16.89 |
| CP+CBD | 55.79 | 53.28 | 2.51 | 38.23 | 50.78 | -12.55 |
| CP+THC | 76.01 | 45.34 | 30.67 | 67.80 | 61.07 | 6.73 |
| THC+CBD+MEL | 85.17 | 69.04 | 16.13 | 53.03 | 63.07 | -10.04 |
| CP+CBD+MEL | 48.49 | 68.14 | -19.65 | 75.22 | 69.96 | 5.26 |
| CP+THC+MEL | 75.90 | 59.88 | 16.02 | 56.41 | 67.46 | -11.05 |
| CP+CBD+THC | 58.78 | 71.33 | -12.55 | 77.91 | 49.97 | 27.94 |
| MEL+CBD+THC+CP | 71.84 | 66.59 | 5.25 | 65.14 | 41.65 | 23.49 |
| Control | 37.18 | 0 | 37.18 | 46.24 | 0 | 46.24 |
|  | **Saos2** | | | **SW1353** | | |
| **% Area** | **Day 1** | **Day 3** | **% Gap** | **Day 1** | **Day 3** | **% Gap** |
| MEL | 74.59 | 40.87 | 33.72 | 96.13 | 35.43 | 60.7 |
| CBD | 92.36 | 69.35 | 23.01 | 39.91 | 51.03 | -11.12 |
| THC | 49.06 | 75.30 | -26.24 | 60.78 | 55.28 | 5.5 |
| CP | 79.95 | 71.96 | 7.99 | 52.11 | 12.63 | 39.48 |
| CBD+MEL | 87.30 | 57.12 | 30.18 | 56.08 | 43.51 | 12.57 |
| THC+MEL | 93.23 | 63.59 | 29.64 | 63.22 | 26.58 | 36.64 |
| CP+MEL | 72.43 | 65.20 | 7.23 | 43.27 | 0 | 43.27 |
| THC+CBD | 72.31 | 59.10 | 13.21 | 59.84 | 17.08 | 42.76 |
| CP+CBD | 85.88 | 54.18 | 31.7 | 71.62 | 60.15 | 11.47 |
| CP+THC | 66.79 | 66.66 | 0.13 | 53.02 | 15.97 | 37.05 |
| THC+CBD+MEL | 55.93 | 45.21 | 10.72 | 45.01 | 35.01 | 10 |
| CP+CBD+MEL | 79.96 | 53.13 | 26.83 | 52.99 | 28.54 | 24.45 |
| CP+THC+MEL | 85.20 | 71.56 | 13.64 | 63.89 | 58.5 | 5.39 |
| CP+CBD+THC | 86.49 | 68.25 | 18.24 | 72.42 | 68.53 | 3.89 |
| MEL+CBD+THC+CP | 69.11 | 70.57 | -1.46 | 31.32 | 52.01 | -20.69 |
| Control | 65.99 | 0 | 65.99 | 46.79 | 0 | 46.79 |

**Supplemental Figure 8.** Effects of tested single applications of MEL, CBD, THC, and CP, and the combination of CBD+MEL on cell migration in Saos2 and SW1353 cell lines. Tests were performed using Saos2 and SW1353 cell lines, showing exponential growth in log phase, and the incubation time was limited to 3 days. % Area calculated with ImageR. Scale 100 µm.

**Supplemental Figure 9.** Effects of tested THC+MEL, CP+MEL, THC+CBD, CP+CBD, and CP+THC combinations on cell migration in Saos2 and SW1353 cell lines. Tests were performed using Saos2 and SW1353 cell lines, showing exponential growth in log phase, and the incubation time was limited to 3 days. % Area calculated with ImageR. Scale 100 µm.

**Supplemental Figure 10.** Effects of tested THC+CBD+MEL, CP+CBD+MEL, CP+THC+MEL, CP+CBD+THC, and MEL+CBD+THC+CP combinations on cell migration in Saos2 and SW1353 cell lines. Tests were performed using Saos2 and SW1353 cell lines, showing exponential growth in log phase, and the incubation time was limited to 3 days. % Area calculated with ImageR. Scale 100 µm.

**Supplemental Figure 11.** Effects of tested single applications of MEL, CBD, THC, and CP, and the combination of CBD+MEL on cell migration in FL and MG63 cell lines. Tests were performed using FL and MG63 cell lines, showing exponential growth in log phase, and the incubation time was limited to 3 days. % Area calculated with ImageR. Scale 100 µm.

**Supplemental Figure 12.** Effects of tested THC+MEL, CP+MEL, THC+CBD, CP+CBD, and CP+THC combinations on cell migration in FL and MG63 cell lines. Tests were performed using FL and MG63 cell lines, showing exponential growth in log phase, and the incubation time was limited to 3 days. % Area calculated with ImageR. Scale 100 µm.

**Supplemental Figure 13.** Effects of tested THC+CBD+MEL, CP+CBD+MEL, CP+THC+MEL, CP+CBD+THC, and MEL+CBD+THC+CP combinations on cell migration in FL and MG63 cell lines. Tests were performed using FL and MG63 cell lines, showing exponential growth in log phase, and the incubation time was limited to 3 days. % Area calculated with ImageR. Scale 100 µm.

**Supplemental Figure 14.** Effects of tested combinations on the MG63 cell line morphology. Tests were performed using the exponentially growing MG63 cell line in log phase, and the incubation time was limited to 24 hours. Scale: 100 µm.

**Supplemental Figure 15.** Effects of tested combinations on the Saos2 cell line morphology. Tests were performed using the exponentially growing Saos2 cell line in log phase, and the incubation time was limited to 24 hours. Scale: 100 µm.

**Supplemental Figure 16.** Effects of tested combinations on SW1353 cell line morphology. Tests were performed using the SW1353 cell line, which exhibits exponential growth in log phase, and the incubation time was limited to 24 hours. Scale: 100 µm.

**Supplemental Figure 17.** Effects of tested combinations on FL cell line morphology. Tests were performed using an exponentially growing FL cell line in log phase, and the incubation time was limited to 24 hours. Scale: 100 µm.

**Supplemental Figure 18.** Control group cells were not treated with any combination but with 0.5% DMSO, which was used to dissolve the test substances. Scale: 100 µm.
